# Rice Phytoalexins: Half a Century of Amazing Discoveries; Part I: Distribution, Biosynthesis, Chemical Synthesis, and Biological Activities

**DOI:** 10.3390/plants12020260

**Published:** 2023-01-05

**Authors:** Alessio Valletta, Lorenzo Maria Iozia, Laura Fattorini, Francesca Leonelli

**Affiliations:** 1 Department of Environmental Biology, Sapienza University of Rome, Piazzale Aldo Moro 5, 00185 Rome, Italy; 2 Department of Chemistry, Sapienza University of Rome, Piazzale Aldo Moro 5, 00185 Rome, Italy

**Keywords:** *Oryza*, rice, phytoalexins, phytocassanes, oryzalexins, momilactones, sakuranetin, phenilammides

## Abstract

Cultivated rice is a staple food for more than half of the world’s population, providing approximately 20% of the world’s food energy needs. A broad spectrum of pathogenic microorganisms causes rice diseases leading to huge yield losses worldwide. Wild and cultivated rice species are known to possess a wide variety of antimicrobial secondary metabolites, known as phytoalexins, which are part of their active defense mechanisms. These compounds are biosynthesized transiently by rice in response to pathogens and certain abiotic stresses. Rice phytoalexins have been intensively studied for over half a century, both for their biological role and their potential application in agronomic and pharmaceutical fields. In recent decades, the growing interest of the research community, combined with advances in chemical, biological, and biomolecular investigation methods, has led to a notable acceleration in the growth of knowledge on rice phytoalexins. This review provides an overview of the knowledge gained in recent decades on the diversity, distribution, biosynthesis, chemical synthesis, and bioactivity of rice phytoalexins, with particular attention to the most recent advances in this research field.

## 1. Introduction

Domesticated rice (*Oryza sativa* L., Poaceae) is one of the widely grown food crops worldwide and is the primary food source in many countries, especially but not only in Asia. Unlike other staple cereal crops, most rice production is used for human consumption in the form of whole-husked grains. In addition to being a primary source of carbohydrates, proteins, and other essential nutrients, rice provides a wide range of bioactive secondary metabolites, including phenolic acids, flavonoids, terpenoids, steroids, and alkaloids. To date, approximately 280 secondary metabolites have been identified in rice. The diversity of secondary metabolites in rice was recently described in an excellent review paper by Wang and co-workers [1].

Like other crop plants, rice is susceptible to various diseases sustained by microorganisms, including viruses, bacteria, and fungi, which cause huge economic losses to farmers [2]. A study published by the International Rice Research Institute (IRRI) reported that, on average, farmers lose 37% of their rice production due to diseases and pests and that these losses can range between 24% and 41%, depending on the production situation [3]. Fungal infections of rice can also pose a significant human health risk due to mycotoxin contamination, especially in geographical areas where rice is a staple food [4].

Plants have evolved multiple defense mechanisms to counteract pathogen attacks, including overproduction of reactive oxygen species (ROS), activation of defense-related genes, synthesis of pathogen-related (PR) proteins, localized strengthening of cell walls, production of pathogen cell wall-degrading enzymes, and accumulation of specialized toxic metabolites, including phytoalexins and phytoanticipins.

The phytoalexin concept was first introduced by Karl O. Müller, who observed that inoculation of potato plants with an incompatible strain of *Phytophthora infestans* induced the biosynthesis of a putative defense compound that conferred resistance to a compatible strain of this phytopathogenic oomycete [5]. In 1940 H. Börger and O. Müller referred to phytoalexins as “chemical compounds produced as a result of invasion of living cells by a parasite” [6]. In the following years, it emerged that the biosynthesis of phytoalexins can also be induced by abiotic stress, such as exposure to UV radiation or heavy metal ions. Furthermore, increasingly sensitive analytical techniques showed that phytoalexins can be produced and/or accumulated even in healthy tissues, albeit at very low levels. Considering these observations, since the 1980s, a broader concept of phytoalexins gained acceptance from the scientific community: “Products of higher plant metabolism, absent from healthy tissues or present only in negligible traces, which accumulate in significant amounts in response to fungal or bacterial challenge” [7].

The concept of phytoanticipins was introduced in 1994 by VanEtten and colleagues [8] with reference to “low molecular weight antimicrobial compounds present in plants before challenge by microorganisms or produced after infection solely from pre-existing constituents”.

According to the above definition, phytoalexins are mainly defined in terms of biosynthesis dynamics and biological role rather than in terms of chemical structure or biosynthetic origin. Indeed, phytoalexins can be biosynthesized through different pathways and thus belong to different classes of secondary metabolites, including terpenoids, phenols, and alkaloids. Sometimes, they are even structurally hybrid compounds whose biosynthesis involves the contribution of multiple biosynthetic pathways [9].

Some of rice’s secondary metabolites, such as momilactone B, exhibit a significant inhibitory activity on seed germination and plant development in addition to playing a key role in plant-pathogen interactions (phytoalexins) and are, therefore, presumed to be involved in plant–plant antagonistic interactions (allelochemicals). The allelopathic activity of natural compounds is attracting growing interest in the research community, stemming from their potential to develop next-generation bioherbicides for use in sustainable weed management [10,11,12,13].

In addition to being key components of the plant defense system, phytoalexins exhibit a wide range of health-promoting biological activities [14,15]. The potential of rice phytoalexins as active ingredients of new-generation antibiotic drugs derives from their proven antiviral [16,17], antitumor [18], antibacterial [19], and antifungal [18,19,20] activity.

Continued investigation of the biosynthesis, diversity, and biological activities of rice phytoalexins will provide foundational knowledge to enable the development of strategies to improve resistance to insect pests, bacterial and fungal diseases, and abiotic stressors, as well as to develop next-generation antimicrobial drugs.

Building on previous experimental and review papers on rice phytoalexins, this review provides a selection of some of the most interesting discoveries that have accumulated over the last few decades regarding the diversity, distribution, biosynthesis, chemical synthesis, and bioactivities of this diverse family of secondary metabolites.

## 2. Diversity and Distribution of Rice Phytoalexins

**Figure 1:**
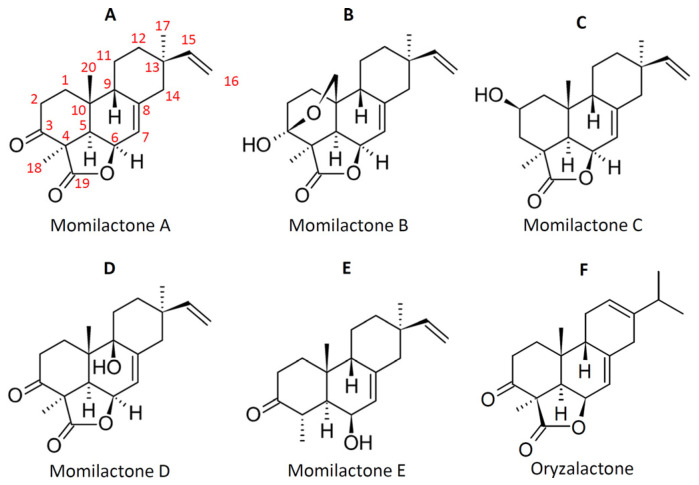
Momilactones so far isolated from rice (**A**–**E**) and oryzalactone (**F**). The dual role of phytoalexins and allelochemicals has been demonstrated for momilactone A and B. Momilactones share a pimarane skeleton, while oryzalactone exhibits an abietane skeleton.

**Figure 2:**
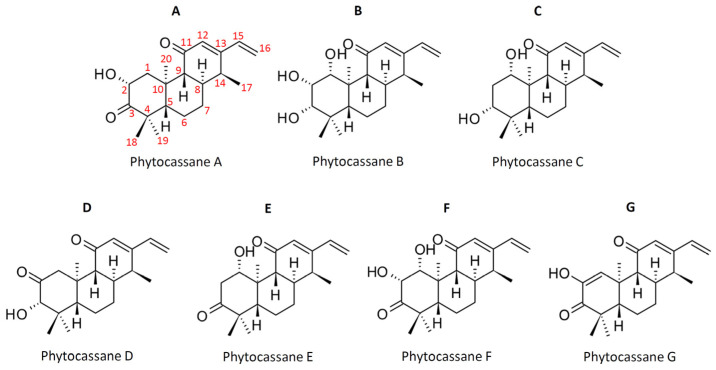
Phytocassanes so far isolated from rice.

**Figure 3:**
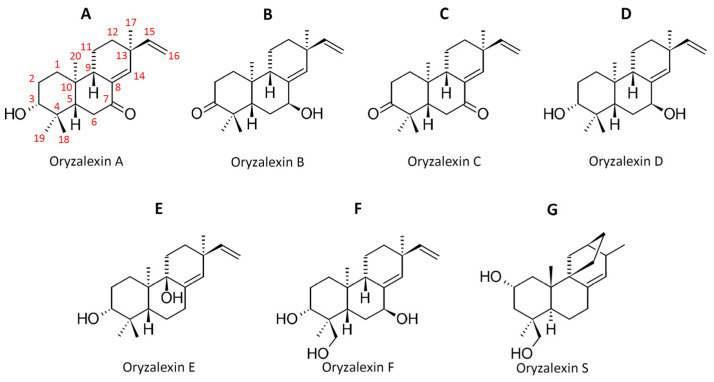
Oryzalexins so far isolated from rice. Despite the name, oryzalexins (**A**–**F**) are distinguished from oryzalexin S both by the chemical structure (*ent*-sandaracopimaradiene- and stemarane-type, respectively) and by the metabolic intermediate from which they derive (*ent*-sandaracopimara-8(14),15 diene and *syn*-stemar-13-ene, respectively; vide infra).

**Figure 4:**
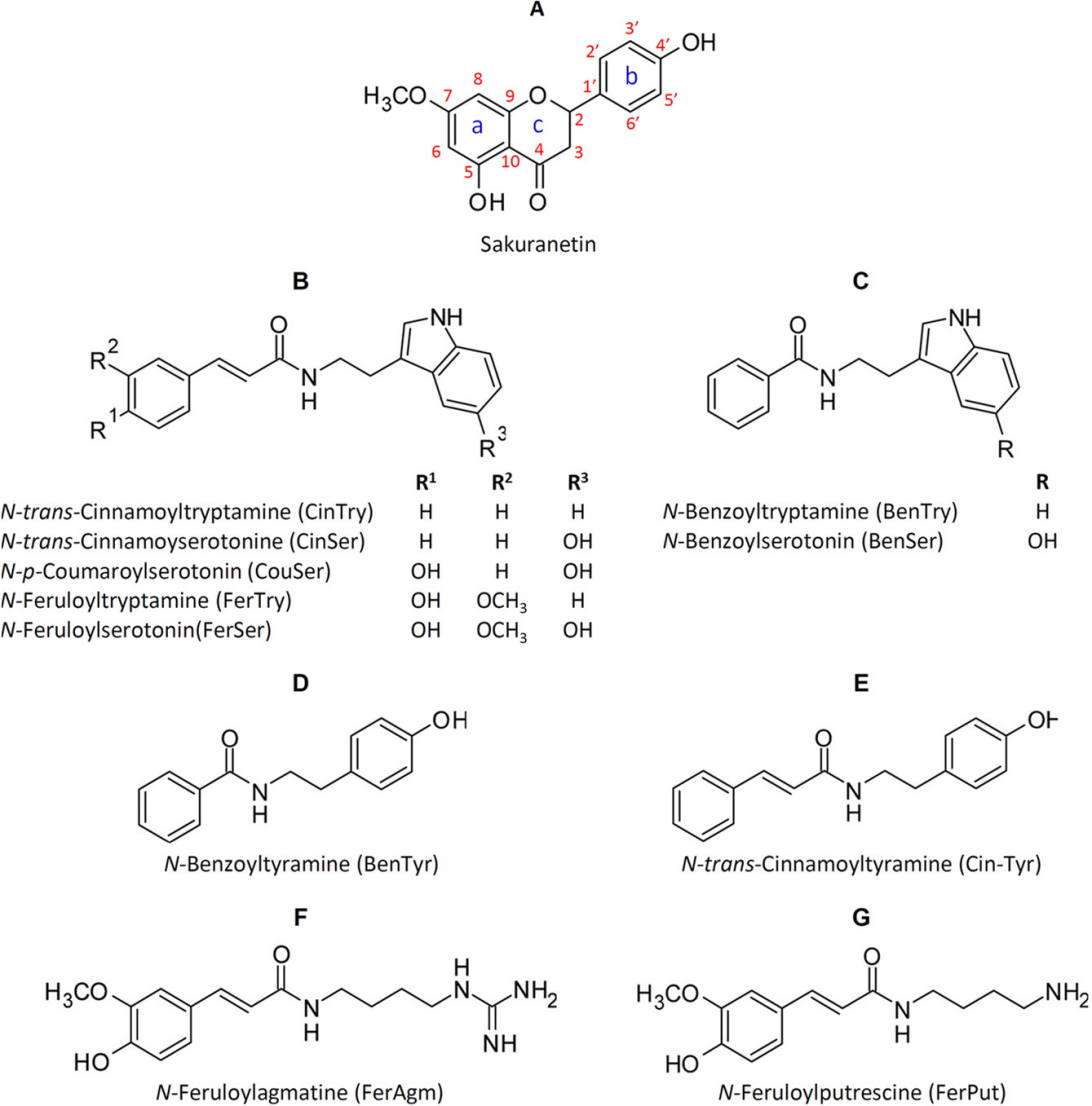
Phenolic compounds that accumulate in rice after exposure to biotic and/or abiotic stresses. (**A**) is a flavanone compound, while (**B**–**G**) are phenylamides.

Since the 1970s, a wide range of phytoalexins has been identified in *Oryza* species. The rice phytoalexins known so far belong to the classes of diterpenes (Figure 1, Figure 2 and Figure 3), flavonoids (Figure 4A), and phenylamides (Figure 4B–G) [9,21].

### 2.1. Diterpenoid Phytoalexins in Rice

Terpenes and terpenoids, also known as isoprenoids, are the largest and most diverse group of natural products, most of which are derived from plants. Of the more than 18,000 plant terpenoids known to date, about 10,000 are diterpenoids (composed of four isoprene units to form a 20-carbon backbone) [22,23]. Monocot diterpenoids almost invariably belong to the large family of labdane-related compounds [24]. This group of diterpenoids is characterized by a labdane-type bicyclic core structure or more complex ring systems derived from labdane-type skeletons, such as abietane, kaurane, pimarane, beyerane, cassane, atisane, stemodane, and manoyl oxide [25]. To date, more than 7000 different labdane-related diterpenoids have been identified in plants [26]. Although they include the plant hormones gibberellins (GAs) as primary metabolites, the vast majority of labdane-related diterpenoids are secondary or rather specialized metabolites, acting as inducible antimicrobial compounds (phytoalexins) and/or plant growth inhibitors involved in antagonistic plant–plant interactions (allelochemicals).

Rice diterpenoid phytoalexins can be classified into four groups based on the structure of their hydrocarbon precursors, namely momilactones A and B [27,28] (Figure 1A,B), phytocassanes A–G [29,30,31] (Figure 2A–G), oryzalexins A–F [32,33,34,35,36,37,38] (Figure 3A–F), and oryzalexin S [39,40] (Figure 3G). Recently, Gu et al. [41] isolated seven diterpenoids from rice hulls, three of which have never been described previously (i.e., 3,20-epoxy-3*α*-hydroxy-8,11,13-abietatrien-7-one; 4,6-epoxy-3*β*-hydroxy-9*β*-pimara-7,15-diene; 2-((*E*)-3-(4-hydroxy-3-methoxyphenyl) allylidene) momilactone A). All isolated compounds showed antifungal activity against four crop pathogenic fungi (i.e., *Magnaporthe grisea*, *Rhizoctonia solani*, *Fusarium oxysporum*, and *Blumeria graminearum*), and phytotoxicity against the major rice weed *Echinochloa crus-galli* (barnyard grass).

#### 2.1.1. Momilactones

Momilactones belong to the small family of (9β-H)-pimarane compounds, which are characterized by a β-orientation of the hydrogen bonded to carbon-9 of the pimarane scaffold [20]. To our knowledge, momilactones A and B (Figure 1A,B) were the first phytoalexins to be characterized by any member of the Poaceae family. They were first identified as plant growth inhibitors from rice seed husks [27] and only later recognized as phytoalexins due to their blast-induced biosynthesis and antifungal activity [28]. The name “momilactone” is the combination of two words, namely *momi*, which is Japanese for rice husk, and lactone, which refers to the chemical structures of momilactones A and B [20]. Other rice momilactones have been identified, whose role as phytoalexins has so far not been demonstrated. Momilactone C (Figure 1C) was isolated in 1976 by Tsunakawa and collaborators [42] as a minor constituent of growth inhibitors from rice seed husks, while momilactones D and E (Figure 1D,E) were isolated in 2015 by Cho and co-workers [43] from rice roots. Momilactone E is a 19-*nor*-(9β-H)-pimarane and does not contain a lactone residue in its molecular structure; therefore, its name is not chemically correct [20].

Although momilactones were initially discovered in cultivated rice (*Oryza sativa*) [27,28], comparative genomic and biochemical studies have recently demonstrated the ability of several wild rice species (i.e., *O. barthii*, *O. brachyantha*, *O. glaberrima*, *O. glumaepatula O. meridionalis*, *O. punctata*, *O. rufipogon*) to biosynthesize momilactones A and B, suggesting that gene clustering for momilactone biosynthesis (see Section 3) had already been accomplished before rice domestication [9,44].

Surprisingly, momilactone biosynthesis has also been found in barnyard grass [45,46], which falls into a separate clade within the Poaceae family. Even more surprising is the identification of momilactones A and B in the moss species *Plagiomnium acutum* (Mniaceae) [47] and *Calohypnum plumiforme* (*Hypnum plumaeforme* prior to 2019, Hypnaceae) [48], which represent a very early diverging lineage of land plants. Ethanol extracts of *C. plumiforme* showed significant growth inhibitory activity against angiosperms, mosses, and liverworts. This suggests that momilactones in bryophytes, as well as in Poaceae, play a role in allelopathy.

Intra- and inter-specific variation in diterpenoid phytoalexin production in rice has not yet been extensively investigated. Recently, Kariya and colleagues [9] analyzed the abundance of diterpenoid phytoalexins in UV-light-irradiated leaves of rice cultivars from the World Rice Core Collection (WRC) (covering a wide range of genetic diversity of rice [49]), and in several wild rice species. Momilactone A was found in most WRC cultivars and wild species, while momilactone B was generally accumulated at lower or undetectable levels. In cultivated rice, the highest content of momilactone A was found in the japonica cultivars (up to 495 nmol g^−1^ FW in ‘Urasan 1’). In wild rice, the greatest accumulation was observed in species with AA and BB genomes (up to 667 nmol g^−1^ FW). In contrast, momilactones were not detected in *O. brachyantha*, which has a FF genome. They also discovered a novel phytoalexin, oryzalactone (Figure 1F), which was only detected in three cultivars in the WRC and in a few strains of wild rice species *O. rufipogon* and *O. meridionalis*. The abietane skeleton of oryzalactone distinguishes it from other momilactones, which are instead characterized by a pimarane skeleton.

#### 2.1.2. Phytocassanes

Several phytocassanes were identified in cultivated [29,30,31] and wild [9] rice. They share an *ent*-cassane-type diterpene skeleton with a C-11 keto group (Figure 2). Modification of the skeleton by the introduction of keto and hydroxy groups increases their chemical variation. Phytocassanes A–D (Figure 2A–D) were first isolated in 1995 by Koga and co-workers [29] from rice stems infected with *Rhizoctonia solani* (the causal agent of rice sheath blight) and from rice leaves infected with *Magnaporthe grisea* (syn. *M. oryzae*, *Pyricularia grisea*/*oryzae*, responsible for rice blast disease). Phytocassanes E and F (Figure 2E,F) were extracted, respectively, from suspension-cultured rice cells elicited with mycelial extracts of the potato pathogenic fungus *Phytophthora infestans* [30] and UV-irradiated rice leaves [31].

It has been shown that phytocassanes, as well as momilactones, accumulate not only in the aerial parts but also in the roots of rice plants. Toyomasu et al. [50] reported that the roots of rice seedlings biosynthesize phytocassanes A–E and momilactones A and B, which are largely released into the environment through exudation. Unlike momilactones, phytocassanes did not show allelopathic activity against dicot seedling growth. Therefore, the authors hypothesized that these compounds might play a role in defense against soil pathogens such as *M. grisea*. Indeed, it has been reported that this fungal pathogen can invade rice roots using a typical root-specific pathway [51].

As mentioned above, the production of diterpenoid phytoalexins in cultivated and wild rice was recently investigated by Kariya and co-workers [9]. All examined cultivars, except for ‘Jinguoyin’, contained phytocassanes A and D, which were also detected in most wild species, especially those with the AA genome (within the genus *Oryza*, species with AA and BB genomes are phylogenetically close to each other and distant from FF [52]). Furthermore, they isolated two undescribed phytoalexins, namely oryzalactone (an isomer of momilactone A) (Figure 1F) and phytocassane G (a di-dehydrogenated phytocassane A) (Figure 2G), from the cultivars ‘Basilanon’ and ‘Jaguary’, respectively. All cultivars in the WRC (except for ‘Jinguoyin’ and ‘Phulba’) showed the phytocassane G-accumulating chemotype, while only three cultivars showed the oryzalactone-accumulating chemotype.

#### 2.1.3. Oryzalexins

Oryzalexins A to F (Figure 3A–F) are *ent*-sandaracopimaradiene-type compounds. Oryzalexin A was first isolated in 1983 by Akatsuka et al. [32] from rice leaves infected with *M. grisea* (Table 1). The in vitro inhibitory activity of oryzalexin A on *M. grisea* conidial germination, with an ED_50_ value of 130 ppm (0.43 mM), was reported by Akatsuka and collaborators [33]. Kariya et al. [53] recently studied the accumulation of oryzalexin A in response to UV light in 69 cultivars from the World Rice Core Collection (WRC) [49] and in 10 strains of the wild species *Oryza rufipogon* (the putative wild ancestor of cultivated rice). They found that only ten of the studied cultivars, belonging to both the *japonica* and *indica* subspecies, produced oryzalexin A. Moreover, both oyzalexin A-producing and non-producing chemotypes were found in *O. rufipogon*, suggesting that the metabolic pathway involved in the biosynthesis of this compound was inherited from an ancestor of *O. rufipogon* and was lost multiple times during evolution. They also reported that, in some cultivars, oryzalexin A accumulation is induced by UV light but not by jasmonic acid (JA), while in others, it is induced by JA but not UV light. This indicates that different signal transduction pathways are induced by UV and JA, and in these cultivars, one of these pathways does not operate [53].

Oryzalexins B–F were identified in cultivated rice subjected to pathogen infection or irradiation by UV light, and their antifungal activity against rice pathogens was ascertained [34,35,36,37,38].

In 1988 Kodama and colleagues [54] found a new antifungal substance that was distinct from the previously known phytoalexins. In 1992 they structurally characterized this new phytoalexin which they named oryzalexin S [55] (Figure 3G). This compound differs from oryzalexins A–F by its stemarane-type skeleton [40].

At present, oryzalexins have only been found in cultivated rice and the related wild species *O. rufipogon* (Table 1).

**Table 1:** Distribution of rice diterpenoid phytoalexins.

Compound	Species (Subspecies, Cultivar)	Part of the Plant	References
Momilactones A and B	*Oryza sativa* L. (subsp. *japonica* cv. Koshihikari)	Seed husks	[27,55]
	*O. sativa* (subsp. *japonica* cvs. Sasashigure; Koshihikari; Haresugata)	Leaves and straw	[28,54,56,57]
	*O. sativa* (subsp. *japonica* cv. Koshihikari)	Root exudates	[58,59,60,61]
	Wild rice species: *Oryza barthii* A. Chev.; *O. brachyantha* A. Chev. et Rhoer.; *O. glaberrima* Steud.; *O. glumaepatula* Steud.; *O. meridionalis* N. Q. Ng; *O. punctata* Kotschy ex Steud.; *O. rufipogon* Griff.	UV-light-irradiated leaves	[9,44]
	*Echinochloa crus-galli* (L.) P.Beauv.	Leaves	[45,46]
	*Calohypnum plumiforme* (Wilson) Jan Kučera & Ignatov (formerly *Hypnum plumaeforme* Wilson)	Aerial parts	[48,62,63,64,65]
	*Plagiomnium acutum* (Lindb.) T. Kop.	Plant material	[47]
Momilactone C	*O. sativa* (subsp. *japonica* cv. Koshihikari)	Seed husks	[42]
	*Pseudoleskeella papillosa* (Lindb.) Kindb	Plant material	[66]
Momilactones D and E	*O. sativa* (subsp. *japonica* cv. Chucheongbyeo)	Roots	[43]
Oryzalactone	*O. sativa* (subsp. *tropical japonica* cv. Jaguary; subsp. *indica* cvs. Local Basmati and Bingala) Wild rice species: *O. rufipogon*; *O. meridionalis*	Leaves irradiated with UV light or inoculated with conidia of *Magnaporthe grisea*	[9]
Phytocassanes A, B, C and D	*O. sativa* (subsp. *japonica* cv. Jukkoku)	Leaves infected with *Magnaporthe grisea* and stems infected with *Rhizoctonia solani*	[29]
Phytocassanes A and D	Wild rice species: *Oryza barthii* A. Chev.; *O. brachyantha* A. Chev. et Rhoer.; *O. glaberrima* Steud.; *O. glumaepatula* Steud.; *O. meridionalis* N. Q. Ng; *O. rufipogon* Griff.	Leaves	[9]
Phytocassane E	*O. sativa* (subsp. *japonica* cv. Koshihikari)	Suspension-cultured rice cells elicited with *Phytophthora infestans* mycelial extract	[30]
Phytocassane F	*O. sativa* (subsp. *japonica* cv. Koshihikari)	UV-irradiated leaves	[31]
Phytocassane G	*O. sativa* (almost all cultivars in the WRC) Wild rice species: *Oryza rufipogon*; *O. glaberrima*; *O. barthii*; *O. glumaepatula*; *O. meridionalis*	UV-irradiated leaves	[9]
Oryzalexin A	*O. sativa* (−)	Leaves infected with *Magnaporthe grisea* (Syn. *Pyricularia oryzae*)	[32]
	*O. sativa* (69 cultivars in the world rice core collection) and *O. rufipogon* (10 strains within the clades, *Or*-I, *Or*-II, or *Or*-III)	Leaves irradiated by UV light, treated with jasmonic acid, or inoculated with conidia of *Bipolaris oryzae*	[53]
Oryzalexins B and C	*O. sativa* (subsp. *japonica* cv. Koshihikari)	Leaves infected with *M. grisea*	[33,34]
Oryzalexins D	*O. sativa* (subsp. *japonica* cv. Koganenishiki)	Leaves infected with *M. grisea*	[36]
Oryzalexin E	*O. sativa* (subsp. *japonica* cv. Nipponbare)	UV-irradiated leaves	[37]
Oryzalexin F	*O. sativa* (subsp. *japonica* cv. Nipponbare)	UV-irradiated leaves	[38]
Oryzalexin S	*O. sativa* (subsp. *japonica* cv. Koshihikari)	UV-irradiated leaves	[39,40,54]

WRC: World Rice Core Collection [49].

### 2.2. Phenolic Phytoalexins in Rice

Sakuranetin (4′,5-dihydroxy-7-methoxyflavanone) (Figure 4A) is the major phenolic phytoalexin in rice. It belongs to the class of flavonoids known as flavanones. Sakuranetin is the O-methylated derivative of the better-known flavanone naringenin, which is predominantly found in some edible fruits such as *Citrus* species, grapefruit, and tomatoes [67]. It was first isolated from the bark of Chinese cherry (*Prunus pseudocerasus*) [68] and wild Himalayan cherry (*Prunus cerasoides* or Prunus puddum) [69] and was later established as a phytoalexin in rice [70]. Over a century of investigations have shown that this flavanone is widely distributed in angiosperm families, generally in its glycosylated form (primarily sakuranin). Some of the sakuranetin-producing plants are listed in Table 2, which is largely based on an excellent review paper recently published by [71] focusing on the pharmacological aspects and distribution of this compound.

The induction of sakuranetin accumulation in response to the rice blast fungus infection [70,72,73], as well as its antimicrobial activity against a wide range of phytopathogenic fungi (e.g., *M. grisea*, *Bipolaris oryzae,* and *R. solani*) and bacteria (e.g., *Burkholderia glumae*, *Xanthomonas oryzae* pv. *Oryzae*, and *X. oryzae* pv. *oryzicola*) strongly suggests its biological role as phytoalexin [73,74].

To understand the role of sakuranetin in rice blast resistance, Hasegawa et al. [73] compared the fungus-responsive characteristics in resistant- and susceptible-type rice lines (IL7 and Nipponbare, respectively). They found that sakuranetin has stronger antifungal activity to blast fungus than momilactone A and that the resistant rice line accumulated sakuranetin in infected regions at an adequate concentration to restrict the fungus, while the amount in the susceptible line was too low to be effective. Moreover, different time-dependent sakuranetin accumulation profiles and hypersensitive responses (HR) were observed in resistant and susceptible lines. Leaves of the resistant line showed HR within three days post-inoculation (dpi) with *M. grisea* spores, with a four-fold increase in sakuranetin accumulation at 4 dpi. Conversely, a susceptible line had an increase in sakuranetin accumulation at 4 dpi but not at 3 dpi, resulting in a large fungus mass without HR.

**Table 2:** Plant species in which sakuranetin has been identified in recent decades.

Species	Family	Part of the Plant	Reference
*Prunus pseudocerasus* Lindl.	Rosaceae	Bark	[68]
*Prunus cerasoides* D.Don (Prunus puddum)	Rosaceae	Bark	[69]
*Eupatorium havanense* Kunth	Asteraceae	Whole plant	[75]
*Ribes nigrum* L.	Grossulariaceae	Leaves	[76]
*Iris milesii* Baker ex Foster	Iridaceae	Rhizomes	[77]
*Artemisia campestris* subsp. *glutinosa* (Gay ex Bess.) Batt.	Asteraceae	Aerial parts	[78]
*Hyptis salzmanii* Benth.	Lamiaceae	Leaves	[79]
*Bonnetia dinizii* Huber	Guttiferae	Wood	[80]
*Primula sieboldii* E. Morren	Primulaceae	Bud exudate	[81]
*Eriodictyon californicum* (Hook. & Arn.) Torr.	Boraginaceae	Leaves	[82]
*Teucrium stocksianum* Boiss.	Lamiaceae	Aerial parts	[83]
*Dodonaea viscosa* Jacq.	Sapindaceae	Aerial parts	[84]
*Xanthorrhoea hastilis* R. Br.	Xanthorrhoeaceae	Dried resin	[85]
*Daphne aurantiaca* Diels.	Thymelaeaceae	Stem bark	[86]
*Dodonaea viscosa* Jacq.	Sapindaceae	Aerial parts	[87]
*Baccharis retusa* DC.	Asteraceae	Twigs	[88]
*Dicerothamnus rhinocerotis* Less.	Asteraceae	Leaves	[89]
*Prunus avium* L.	Rosaceae	Sweet cherry	[90]
*Viscum album* L.	Santalaceae	Tinctures	[91]

As will be discussed later, the direct precursor of sakuranetin is supposed to be naringenin. Sakuranetin showed significantly higher antifungal activity than naringenin against *M. grisea* [70,92]. Interestingly, sakuranetin can be detoxified into naringenin and sternbin (another flavanon compound) by *M. grisea* [92] and into naringenin, sakuranetin-4′-*O*-β-d-xylopyranoside, and naringenin-7-*O*-β-d-xylopyranoside by the rice sheath blight fungus *R. solani* [93].

Until recently, sakuranetin was considered the only phenol phytoalexin in rice. However, recent studies have shown that several phenylamides (e.g., *N*-cinnamoyltyramine, *N*-benzoyltryptamine, *N*-feruloyltryptamine, *N*-cinnamoyltryptamine, *N*-feruloyltyramine, *N-p*-coumaroylserotonin, and *N*-feruloylserotonin) (Figure 4) are produced by rice in response to biotic or abiotic stress. Phenylamides are secondary metabolites widely distributed in plants resulting from the conjugation of mono- or polyamines with aromatic acids, like caffeic, ferulic, and *p*-coumaric acids [94]. Amine moieties found in rice phenylamides include the arylmonoamines tyramine, tryptamine, and serotonin [21].

Some of the phenylamides found in rice have shown significant antimicrobial activity against several bacterial and fungal pathogens such as *Burkholderia glumae*, *Xanthomonas oryzae*, *Magnaporthe grisea,* and *Cochliobolus miyabeanus* [74,95,96]. This observation strongly suggests that, together with sakuranetin, phenylamides are members of phenolic phytoalexins in rice [21,96].

Ishihara and co-workers [97,98] identified several phenylamides, including *N-p*-coumaroylserotonin (CouSer), *N*-feruloyltryptamine (FerTry), and *N*-feruloylserotonin (FerSer), in rice leaves infected with the blast fungus *M. oryzae* and the rice brown spot fungus *B. oryzae.* Quinet and colleagues [99] observed that salt stress in leaves of the salt-resistant rice cultivar Pokkali led to an increase in putrescine amides, which are presumably conjugated with phenolic acids or other low molecular weight compounds. UV-induced accumulation of *N*-benzoyltryptamine (BenTry) and *N*-*trans*-cinnamoyltyramine (CinTyr) in rice was also observed by Park and colleagues [100]. *N*-Benzoyltyramine (BenTyr) was isolated from rice leaves exposed to UV radiation by Horie et al. [95]. The production of this compound was also induced by inoculation with *M. oryzae*. However, unlike CinTyr and BenTry, BenTyr has shown negligible antifungal activity against this pathogen, and further antimicrobial testing is needed to investigate its possible role in plant defense against pathogen attack. To provide a full picture of inducible phenylamides in rice, Morimoto and co-workers [96] monitored the accumulation of 25 phenylamides in rice leaves after infection with *C. miyabeanus* and *X. oryzae*. Both pathogens caused significant increases in phenylamide accumulation, although the greatest effects were recorded after *C. miyabeanus* infection, which mainly induced the accumulation *N*-feruloylputrescine (FerPut), BenTry, and *N*-benzoylserotonin (BenSer), followed by BenTry, *N-trans*-cinnamoylserotonin (CinSer), CouSer, FerSer, CinTyr, *N*-feruloylagmatine (FerAgm), and *N-trans*-cinnamoyltryptamine (CinTry). Phenylamide accumulation has also been induced by treatment with different hormones, including jasmonic acid, salicylic acid, and 6-benzylaminopurine.

## 3. Rice Phytoalexin Biosynthesis

### 3.1. Biosynthesis of Rice Diterpenoid Phytoalexins

**Figure 5:**
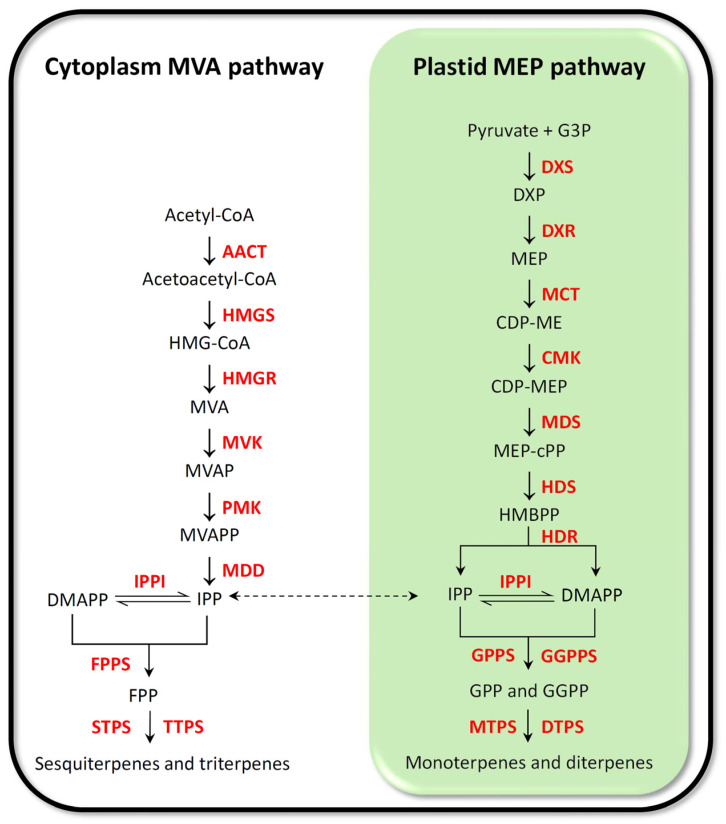
Biosynthesis of IPP and DMAPP through MVA and MEP pathway in plants. Although IPP and DMAPP act as precursors for all terpenes, sesqui-, and triterpenes biosynthesis typically is fed by the cytosolic MEV pathway, while mono-, di-, and tetraterpenes biosynthesis typically is fed by the plastidial MEP pathway. AACT: acetoacetyl-CoA thiolase (EC 2.3.1.9); CMK: 4-(cytidine 5′-diphospho)-2-C-methyl-D-erythritol kinase (EC 2.7.1.148); DMAPP: dimethylallyl diphosphate; DPTS: diterpene synthase (E.C. 4.2.3.x); DXR: 1-deoxy-D-xylulose 5-phosphate reductoisomerase (EC 1.1.1.267); DXS: 1-deoxy-D-xylulose 5-phosphate synthase (EC 2.2.1.7); FDP: farnesyl diphosphate; FPPS: farnesyl diphosphate synthase (EC 2.5.1.10); G3P: D-glyceraldehyde 3-phosphate; GPP: geranyl diphosphate; GPPS: geranyl diphosphate synthase (EC 2.5.1.29); GGPPS: geranylgeranyl diphosphate synthase (EC 2.5.1.29); HDR: (E)-4-hydroxy-3-methylbut-2-enyl diphosphate reductase; HDS: (E)-4-hydroxy-3-methylbut-2-enyl diphosphate synthase (EC 1.17.1.2); HMGR: 3-hydroxy-3-methylglutaryl-CoA reductase (EC 1.17.1.2); HMGS: 3-hydroxy-3-methylglutaryl-CoA synthase (EC 2.3.3.10); IPPI: isopentenyl diphosphate Δ-isomerase (EC 5.3.3.2); IPP: isopentenyl diphosphate; MCT: 2-C-methyl-d-erythritol 4-phosphate cytidylyltransferase (EC 2.7.7.60); MDD: diphosphomevalonate decarboxylase (EC 4.1.1.33); MDS: 2-C-methyl-D-erythritol 2,4-cyclodiphosphate synthase (EC 4.6.1.12); MTPS: monoterpene synthase (EC:4.2.3.-); MVK: mevalonate kinase (EC 2.7.1.185); MVAP: mevalonate 5-phosphate; MVAPP: mevalonate diphosphate; PMK: phosphomevalonate kinase (EC 2.7.4.2); STPS: sesquiterpene synthase (EC 4.2.3.49; 4.2.3.47; 3.1.7.6); TPS: terpene synthase (EC 4.2.3.47); TTPS: triterpene synthase (EC 5.4.99.-) (adapted from [107]).

All isoprenoids are derived from the common five-carbon precursor isopentenyl diphosphate (IPP) and its allylic isomer dimethylallyl diphosphate (DMAPP). In plants, IPP and DMAPP are biosynthesized through two independent pathways: the mevalonate (MVA) pathway, which occurs in the cytosol, and the methylerythritol-4-phosphate (MEP) pathway, localized in the chloroplast (Figure 5). Certain isoprenoids, such as monoterpenes, diterpenes, carotenoids, tocopherols, and the side chains of chlorophylls, are formed via the MEP pathway, while others, such as phytosterols, sesquiterpenes, triterpenes, and the side chain of ubiquinone are biosynthesized via the MVA pathway [101]. Several lines of evidence suggest that the MEP pathway is involved in the biosynthesis of diterpenoids [102,103], although some MEP-MVA crosstalk could not be ruled out [104,105]. Microarray analysis showed a clear correlation between the expression of MEP pathway genes and the accumulation of diterpenoid phytoalexins in elicitor-treated suspension cells [102].

Isopentenyl diphosphate Δ-isomerase (IPPI) catalyzes the reversible conversion of IPP to DMAPP. To convert IPP to DMAPP in the cytoplasm, IPPI is required, and in the absence of this enzyme, the MEP pathway is blocked. In contrast, IPPI is not strictly essential in plastids where a mixture of DMAPP and IPP is produced from 4-hydroxy-3-methylbut-2-enyl diphosphate (HMBPP) by the last enzyme in the MEP pathway HMBPP reductase (HDR) [106]. Two IPPI isoforms, OsIPPI1 and OsIPPI2, have been identified in rice. The subcellular localization of OsIPPI1 and OsIPPI2 was recently investigated by Jin et al. [101] by constitutively expressing these enzymes fused to synthetic green fluorescent protein (sGFP). Although both isoforms were detected in the endoplasmic reticulum (ER), mitochondria and peroxisomes, only OsIPPI2 was identified in plastids. However, *OsIPPI2* gene expression did not correlate with chlorophyll or carotenoid accumulation in plastids, suggesting that it may be a redundant component of the MEP pathway. Colocalization of both OsIPPI1 and OsIPPI2 in the ER suggests that DMAPP may be synthesized *de novo* in this compartment in rice [101].

**Figure 6:**
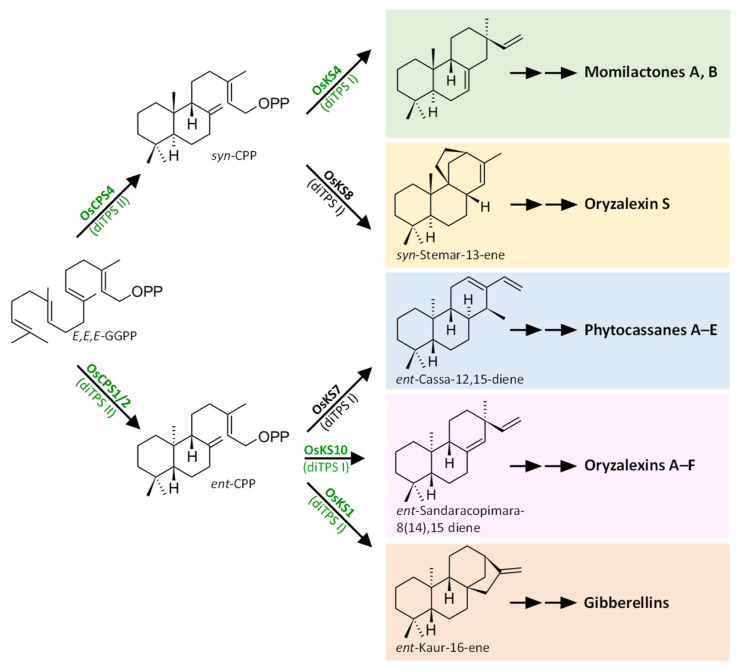
Proposed pathway for the biosynthesis of rice diterpenoid phytoalexins and gibberellins. The enzymes whose chloroplast localization is established are written in green. CPP: copalyl diphosphate; diTPS I: class I diterpene synthase; diTPS II: class II diterpene synthase; GGPP: geranylgeranyl diphosphate; OsCPS1: *ent*-copalyl diphosphate synthase (EC 5.5.1.13); OsCPS2 (OsCyc2): *ent*-copalyl diphosphate synthase (EC 5.5.1.13); OsCPS4 (OsCyc1): *syn*-copalyl diphosphate synthase (EC 5.5.1.14); OsKS1: *ent*-kaur-16-ene synthase (EC 4.2.3.19); OsKS4 (OsKSL4): *syn*-pimara-7,15-diene synthase (EC 4.2.3.35); OsKS7 (OsKSL7; OsDTC1): *ent*-cassa-12,15-diene synthase (EC 4.2.3.28); OsKS8 (OsKSL8; OsK8; OsDTC2): stemar-13-ene synthase (EC 4.2.3.33); OsKS10 (OsKSL10): *ent*-sandaracopimara-8(14),15-diene synthase (EC 4.2.3.29) (adapted from [110]).

Higher molecular weight isoprenoids are synthesized by the addition of one or more IPP units to a DMAPP, with a simultaneous release of the pyrophosphate anion (PPi). DMAPP is mainly used as a chemically active substrate, which is extended by the addition of IPP units to short-chain prenyl diphosphates such as geranyl diphosphate (GPP, C10), farnesyl diphosphate (FPP, C15), and geranylgeranyl diphosphate (GGPP, C20) (Figure 5). GGPP is the central precursor of all diterpenoids, including rice diterpenoid phytoalexins (Figure 6). The IDS responsible for GGPP biosynthesis is GGPP synthase (GGPPS).

The structural diversity of labdane-type diterpenoids arises from the pairwise activity of class I and class II diterpene synthases (diTPSs) which act sequentially to convert GGPP into distinct diterpenoid scaffolds [24,26,108] (Figure 6). Class II diTPSs catalyze the first step of rice diterpenoid biosynthesis, consisting of the conversion of (*E*,*E*,*E*)-GGPP into the bicyclic prenyl diphosphate intermediates (*5S,9S,10R*)-copalyl diphosphate (*syn*-CPP) and (*5R*,*9S*,*10S*)-copalyl diphosphate (*ent*-CPP). Two class II diTPSs belonging to the TPS-c subfamily [24,109] are responsible for the biosynthesis of *syn*-CDP and *ent*-CDP, i.e., OsCPS4 and OsCPS1/2, respectively. Subsequently, class I diTPSs convert *syn*-CPP and *ent*-CPP via ionization of the diphosphate substituent and several cyclization and rearrangement reactions downstream of the carbocation intermediate [26].

Many genes encoding enzymes involved in the biosynthesis of rice diterpenoid phytoalexins are arranged as biosynthetic gene clusters (BGCs). A BGC is made up of three or more non-homologous genes encoding enzymes involved in the same biosynthetic pathway and located close to each other on the same chromosome. The clustering of genes encoding protein complexes has been proposed to be a useful strategy for coordinating the regulation of component genes and providing an optimal proportion of gene products. Furthermore, the grouping of biosynthetic pathways could help reduce autotoxicity caused by the accumulation of intermediates. The co-inheritance of entire biosynthetic pathways promotes the evolution of common gene expression regulation mechanisms, accelerating responses to environmental conditions and maximizing plant fitness [111,112,113]. Gene clusters associated with the biosynthesis of secondary metabolites have been found in several plant species: benzoxazinone BCG in maize [114], avenacin BCG in oats [115], thalianol, and marneral BGC in Arabidopsis [116,117], potential BGCs in cucumber [118], as well as a new momilactone BGC in barnyard grass [45].

**Figure 7:**
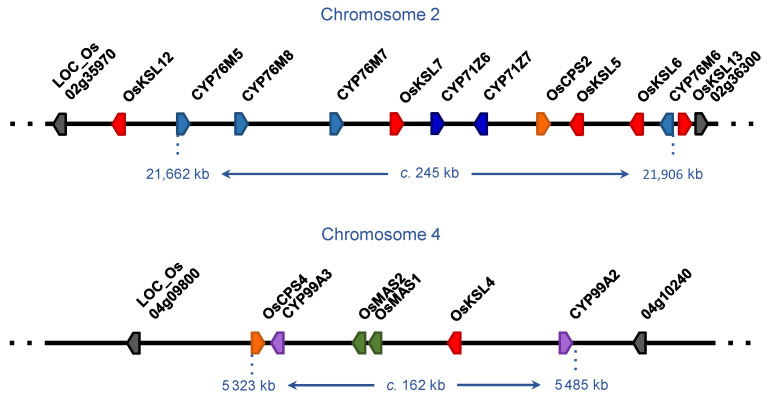
Schematic diagram of loci corresponding to genes involved in diterpene biosynthesis in rice. The biosynthetic gene clusters on chromosome 2 (c2BGC) and chromosome 4 (c4BGC) are located between LOC_Os02g35970 and LOC_Os02g36300 and between LOC_Os04g09800 and LOC_Os04g10240, respectively (adapted from [44,119,126]).

At least two BGCs associated with rice diterpenoid phytoalexin biosynthesis have been identified in the genome of cultivated rice. They are c4BGC, involved in momilactone A biosynthesis on chromosome 4, and c2BGC, associated with phytocassane biosynthesis on chromosome 2 [44,113,119,120,121] (Figure 7). Several lines of evidence suggest that c2BGC plays a more general biosynthetic role, e.g., the production of oryzalexins and oryzalides [122,123,124]. Furthermore, it has been shown that the biosynthesis of momilactones requires the contribution of genes belonging to both c4BGC and non-clustered genes located on different chromosomes [125].

#### 3.1.1. Biosynthesis of Momilactones

**Figure 8:**
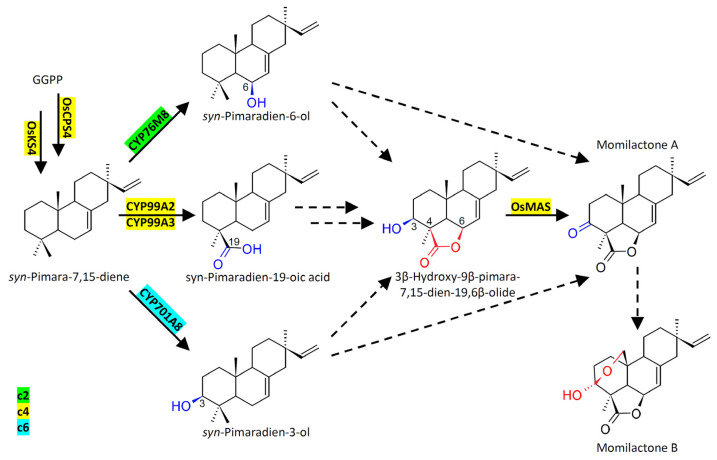
Proposed pathway for the biosynthesis of momilactones A and B. Chemical functions in blue indicate characterized reactions, while predicted functionalities are indicated in red. Dashed arrows indicate missing steps. Enzymes encoded by genes on chromosome 2, 4, and 6 (c2, c4, c6) are highlighted in green, yellow, and blue, respectively. GGPP: geranyl geranyl diphosphate; OsMAS: rice momilactone A synthase; OsKS4 (OsKSL4): *syn*-pimara-7,15-diene synthase (EC 4.2.3.35); OsCPS4 (OsCyc1): *syn*-copalyl diphosphate synthase (EC 5.5.1.14); CYP76M8: oryzalexin D synthase (EC 1.14.14.112; 1.14.14.123); CYP99A3: 9-beta-pimara-7,15-diene oxidase (EC 1.14.14.111); CYP99A2: cytochrome P450 99A2 (EC 1.14.-.-); CYP701A8: *ent*-sandaracopimaradiene 3-hydroxylase (EC 1.14.14.70) (adapted from [128]).

Although several enzymes involved in momilactone biosynthesis have been identified, the order of known steps and the reconstitution of multiple characterized enzymes in vivo remains elusive [127,128]. To date, the diterpene synthases OsCPS4 and OsKS4 responsible for the tricyclic momilactone scaffold formation (Figure 6) have been characterized [129,130,131,132]. These enzymes are encoded by genes clustered with genes encoding cytochrome P450 enzymes (CYPs), *CYP99A2* and *CYP99A3*, and a short-chain dehydrogenase reductase (SDR), *OsMAS*, in the biosynthetic gene cluster on rice chromosome 4 (c4BGC) [125] (Figure 7). This BGC has also been found in the genomes of other *Oryza* species with AA-genome, namely *O. rufipogon* and *O. punctata*, as well as in the distantly related momilactone-producing species *Echinochloa crus-galli* and *Calohypnum plumiforme* [45,113,133] (Table 1). CYP99A3 and/or CYP99A3 together with two non-clustered CYPs, CYP701A8, and CYP76M8 (coded by genes located on chromosome 6 and 2, respectively), oxidize the diterpene scaffold *syn*-pimaradiene at different positions [134,135] (Figure 8). The order in which these enzymes act to form a skeleton oxidized at different positions has not yet been fully understood [124,134,135]. In vitro tests showed that SDR encoded by *OsMAS* genes is capable of oxidizing 3β-hydroxy-9β-pimara-7,15-dien-19,6β-olide to form momilactone A [120].

More details on the biosynthesis and biogenesis of momilactones and related rice diterpenoids can be found in two excellent reviews recently published by Zhao et al. [20] and Serra Serra et al. [12].

#### 3.1.2. Biosynthesis of Phytocassanes

As mentioned above, at least two biosynthetic gene clusters (BGCs) are involved in rice diterpenoid phytoalexin biosynthesis, c2BGC and c4BGC, which are nominally associated with phytocassane and momilactone production, respectively [119,120] (Figure 7). It has also been reported that the production of momilactone A requires the intervention of enzymes encoded by genes not belonging to c4BGC, namely CYP701A8, responsible for hydroxylation at C3β [135] and CYP76M8 for hydroxylation at C6β [124] (Figure 8). It should be noted that *CYP76M8* belongs to c2BGC, which also contains the gene *OsKLS7* involved in the first step of phytocassane biosynthesis [136], as well as the upstream acting genes *OsCPS1* and *OsCPS2* [131,137] (Figure 6, Figure 7 and Figure 8).

**Figure 9:**
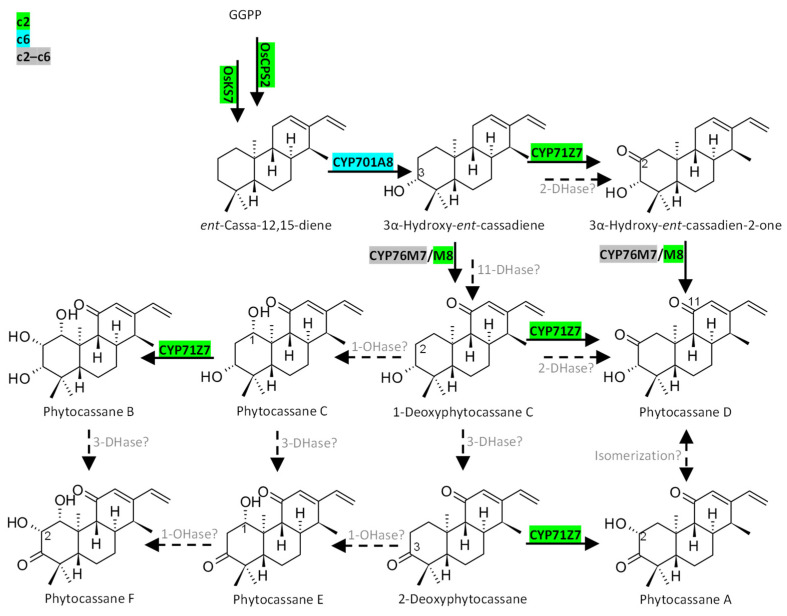
Proposed pathway for the biosynthesis of phytocassanes. The green highlight indicates enzymes encoded by genes belong to the biosynthetic gene cluster on chromosome 2 (c2BGC). Enzymes encoded by genes located on chromosomes 6 and in both 2 and 6 (c6 and c2–c6) are highlighted in blue and grey, respectively. GGPP: geranyl geranyl diphosphate; OsKS7 (OsKSL7; OsDTC1): *ent*-cassa-12,15-diene synthase (EC 4.2.3.28); OsCPS2 (OsCyc2): *ent*-copalyl diphosphate synthase (EC 5.5.1.13); CYP76M7: *ent*-cassadiene C11-alpha-hydroxylase 1 (EC 1.14.14.112); CYP76M8: oryzalexin D synthase (EC 1.14.14.112; 1.14.14.123); CYP701A8: *ent*-sandaracopimaradiene 3-hydroxylase (EC 1.14.14.70); C71Z7: *ent*-cassadiene hydroxylase (EC 1.14.14.69) (adapted from [138]).

Six cytochrome P450 enzymes (CYP71Z6 and Z7, CYP76M5 to M8) encoded by genes on rice chromosome 2 contribute to phytocassane biosynthesis [138]. In vitro enzyme assay systems have been extensively used to explore the biological role of these P450s in phytocassane biosynthesis. However, little information is available regarding the in planta contribution of relevant P450 genes. Recently, Ye et al. [138] investigated their involvement in phytocassane production in planta by exploiting loss-of-function rice plants defective in each P450 gene. By characterizing CYP76M7/M8- and CYP71Z7-suppressed plants, they proposed a phytocassane biosynthetic pathway and identified novel candidate intermediates (Figure 9). They speculated that, in planta, CYP76M7/M8 are responsible for C11α-hydroxylation of 3-hydroxy-*ent*-cassadiene and CYP71Z7 is involved in C2-hydroxylation of phytocassanes. Further studies are needed to confirm this hypothesis and fill gaps in the proposed pathway through the discovery of currently unknown enzymes that are likely encoded by genes outside of the cluster.

Interestingly, phytocassane BGC has also been found in non-*Oryza* species, such as Chinese rice (*Zizania latifolia*), with complementary subclusters separated on chromosomes 8 and 10 [139].

#### 3.1.3. Biosynthesis of Oryzalexins

Despite their names, oryzalexins A–F are distinguished from oryzalexin S both structurally and biosynthetically (Figure 3 and Figure 6). Oryzalexins A–F are *ent*-sandaracopimaradiene-type compounds derived from *ent*-sandaracopimara-8(14),15-diene, while oryzalexin S is a stemarane-type compound derived from *syn*-stemar-13-ene.

**Figure 10:**
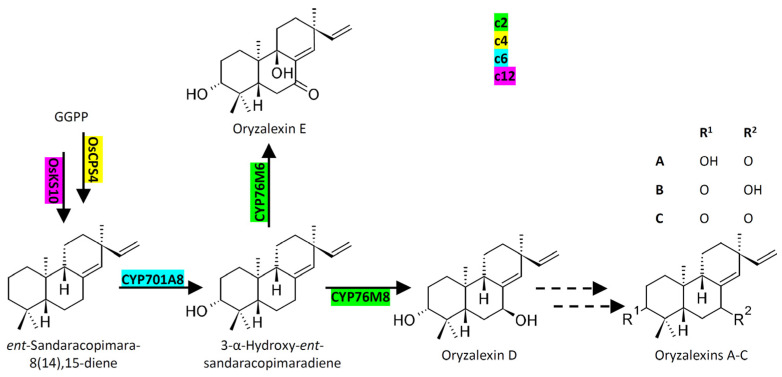
Proposed pathway for the biosynthesis of oryzalexins A–E. Enzymes encoded by genes located on chromosomes 2, 4, 6, and 12 (c2, c4, c6, c12) are highlighted in green, yellow, blue, and pink, respectively. A, B, C: oryzalexin A, B, and C, respectively. CYP701A8: *ent*-sandaracopimaradiene 3-hydroxylase (EC 1.14.14.70); CYP76M6: oryzalexin E synthase (EC 1.14.14.122); CYP76M8: oryzalexin D synthase (EC 1.14.14.112; 1.14.14.123); GGPP: geranyl geranyl diphosphate; OsCPS4: *syn*-copalyl diphosphate synthase (EC 5.5.1.14); OsKS10: *ent*-sandaracopimara-8(14),15-diene synthase (EC 4.2.3.29) [123,138].

As shown in Figure 10, hydroxylation of *ent*-sandaracopimara-8(14),15-diene catalyzed by CYP701A8 results in the formation of 3α-hydroxy-*ent*-sandaracopimaradiene, the putative precursor of oryzalexins A–E [124]. This precursor is then converted to oryzalexins D and E by the P450 enzymes CYP76M8 and CYP76M6, respectively [123]. Several short-chain oxidoreductases oxidize oryzalexin D at different positions to form oryzalexins A–C [135].

In contrast to their *ent*-sandaracopimaradiene-derived counterparts, little information is available on the biosynthesis of oryzalexin S. The biosynthetic pathway of this compound branches when GGPP is converted into *syn*-CDP by OsCPS4 (Figure 6). At that point, OsKS8 converts it into *syn*-stemar-13-ene [24,26,108]. To date, the last steps concerning the addition of the hydroxyl groups to C2β and to the methyl substituent in C4β, which characterize oryzalexin S, have not been resolved.

### 3.2. Biosynthesis of Rice Flavonoid Phytoalexins

**Figure 11:**
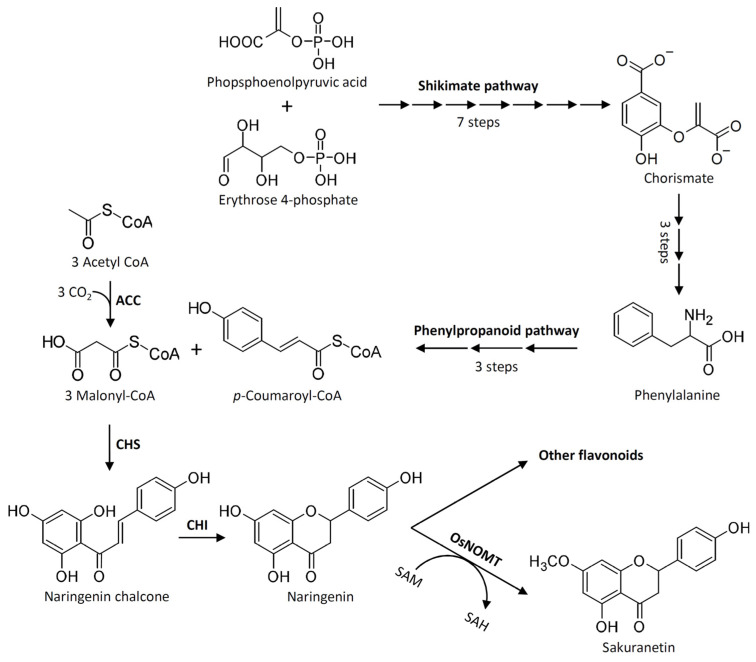
Biosynthesis of sakuranetin. ACC: acetyl-CoA carboxylase (EC 6.4.1.2); CHI: chalcone isomerase (EC 5.5.1.6); CHS: chalcone synthase (EC 2.3.1.74); OsNOMT: naringenin 7-O-methyltransferase (EC 2.1.1.232); SAH: adenosyl-L-homocysteine; SAM: S-adenosyl-L-methionine. Multiple arrows indicate multiple biosynthetic steps, while direct synthesis is indicated by single arrows.

Sakuranetin is a flavonoid belonging to the group of methoxylated flavanones. Like most plant-based flavonoids, sakuranetin derives from the aromatic amino acid phenylalanine (Phe). In plants, the precursor of aromatic amino acids is chorismate, the end product of the shikimate pathway (Figure 11). Phe is then converted to *p*-coumaroyl-CoA via the phenylpropanoid pathway. Chalcone synthase (CHS) catalyzes the condensation of three malonyl-CoA molecules with one *p*-coumaroyl-CoA molecule to form the open-chain flavonoid naringenin chalcone, which is converted to naringenin by chalcone isomerase (CHI). Naringenin is then transformed into sakuranetin by S-adenosyl-l-methionine-dependent naringenin 7-O-methyltransferase (OsNOMT). OsNOMT can either be activated by UV or fungal infection [70,72]. The 7-O-methylation appears to be crucial for the antifungal activity of sakuranetin, as naringenin itself does not exhibit significant toxic effects against rice fungal pathogens such as *M. grisea* [70]. Since naringenin is the common biosynthetic intermediate for several flavonoids, OsNOMT is a key player at the branch point between flavonoid and sakuranetin biosynthesis (Figure 11). OsNOMT was purified from UV-treated leaves of the *oscomt1* rice mutant, and the corresponding gene was identified by Shimizu et al. [140]. Several putative flavonoid O-methyltransferase genes (*OMTs*) have been identified in the rice genome, and thus others may be involved in sakuranetin biosynthesis.

**Figure 12:**
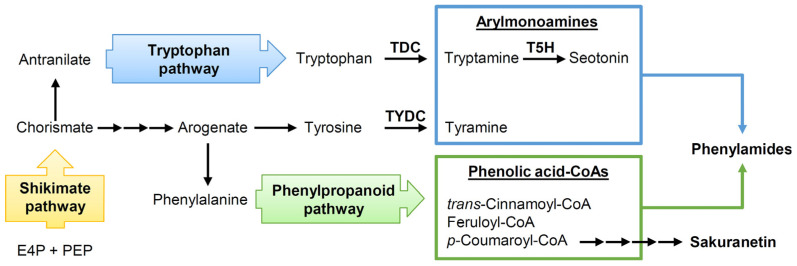
Biosynthesis of phenylamides and sakuranetin. Multiple arrows indicate multiple enzymatic steps. E4P: erythrose 4-phosphate; PEP: phosphoenol pyruvate; T5H: tryptamine 5-hydroxylase (EC 1.14.-.-); TDC: L-tryptophan decarboxylase (aromatic-L-amino-acid decarboxylase, EC 4.1.1.28); TYDC: L-tyrosine decarboxylase (EC 4.1.1.25) (adapted from [21]).

Phenylamides are formed by the conjugation of phenolic acid-CoAs (e.g., *p*-coumaroyl-CoA, *trans*-cinnamoyl-CoA, and feruloyl-CoA) with arylamines [94,100,141] (Figure 12). Tyramine, tryptamine, and its derivative serotonin are the aryl monoamines found in rice phenylamide phytoalexins [74,97,98,100]. Tyramine and tryptamine result from the decarboxylation of the aromatic amino acids tryptophan (Try) and tyrosine (Tyr) catalyzed by Try decarboxylase (TDC) and Tyr decarboxylase (TYDC), respectively. Tyr is synthesized downstream of the shikimate pathway, which also provides precursors to the other aromatic amino acids Phe and Try [142]. Serotonin (5-hydroxytryptamine) is synthesized from tryptamine by tryptamine 5-hydroxylase (T5H). Additional details regarding the pathways involved in the biosynthesis of rice phenolic phytoalexins and their induction by abiotic and biotic stress can be found in Cho and Lee [21].

## 4. Chemical Synthesis of Rice Phytoalexins

Several research groups have tried their hand at the chemical synthesis of rice phytoalexins. However, only limited data is currently available on this topic (Table 3), and the main source of these compounds is still natural. From a general point of view, organic synthesis is a very valuable tool in the study of molecules with a complex skeleton. Indeed, it is possible to use chemical synthesis not only to establish the molecular structure, which often cannot be elucidated with X-ray analysis due to the small amount of the naturally occurring compound, but also to produce bioactive compounds in quantity adequate to carry out a structure–activity relationship study. Furthermore, organic synthesis allows for the development of efficient synthetic strategies, useful for the preparation of derivatives that could eventually result in more potent bioactivity. Despite all these potentials, the *de novo* synthesis of rice phytoalexins (with the exception of sakuranetins, the smaller and least complex ones) cannot be considered, up to now, a method to supply these molecules to the agrochemical industry, due to the large number of steps required by the developed synthetic plans that result in an overall yield.

### 4.1. Momilactones Chemical Synthesis

The only momilactone that has been synthesized is (±)-momilactone A (Table 3, entry 1) [143]. The preparation of this compound was preceded by several synthetic studies on the pimarane skeleton that were reported, along with its synthesis, in a recent review [156].

### 4.2. Phytocassane Chemical Synthesis

**Scheme 1:**
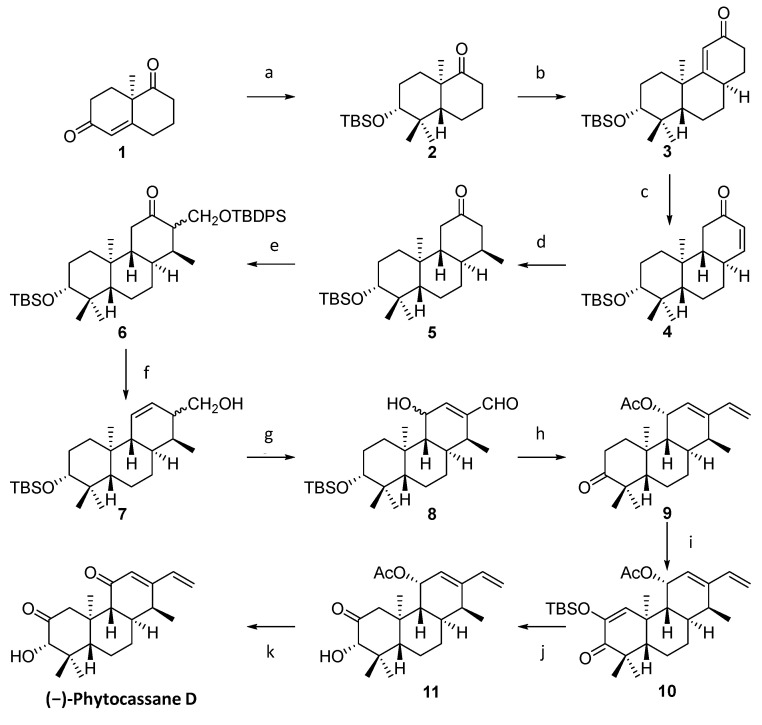
(−)-Phytocassane D chemical synthesis [144]. Reagents and conditions: (a) (i) 2,2-dimethyl-1,3-propandiol, BF_3_.Et_2_O; (ii) PhSH, aq. CH_2_O, Et_3_N, EtOH; (iii) Li, liq. NH_3_, H_2_O, THF, then MeI, THF; (iv) LiAlH_4_, THF; (v) aq. HCl, THF; (vi) TBSCl, imidazole, DMF (40%, 6 steps). (b) (i) NaH, HCO_2_Et, THF, PhMe; (ii) MeCOCH=CH_2_, Et_3_N; (iii) NaOMe, MeOH (78%, 3 steps). (c) (i) Li, liq. NH_3_, EtOH, THF; (ii) PCC, 3-Å MS, CH_2_Cl_2_; (iii) KH, PhSO_2_Me, THF; (iv) CaCO_3_, PhMe, heat (77%, 4 steps). (d) (i) Me_2_CuLi, Et_2_O; (ii) diastereoisomer separation (47%). (e) (i) NaH, HCO_2_Et, MeOH; (ii) NaBH_4_, THF, MeOH; (iii) TBDPSCl, imidazole, DMF; (iv) PCC, 4-Å MS, CH_2_Cl_2_ (57%, 4 steps). (f) (i) TsNHNH_2_, MgSO_4_, PPTS, THF; (ii) excess LDA, THF then quenched with aq. NH_4_Cl; (iii) TBAF, THF, room temp. (68%, 3 steps). (g) (i) MCPBA, NaHCO_3_, CHCl_3_; (ii) Dess–Martin periodinane, CH_2_Cl_2_; (iii) pyrrolidine, Et_2_O (67%, 3 steps). (h) (i) Ph_3_P=CH_2_, THF; (ii) Ac_2_O, DMAP, C_5_H_5_N; (iii) TBAF, THF, 50–60 °C; (iv) PCC, 4-Å MS, CH_2_Cl_2_ (59%, 4 steps). (i) (i) LiHMDS, TMSCl, THF; (ii) MCPBA, NaHCO_3_, hexane; (iii) (CO_2_H)_2_, MeOH; (iv) Dess–Martin periodinane, CH_2_Cl_2_; (v) TBSCl, imidazole, DMF (25%, 5 steps). (l) LiAlH_4_, THF, then aq. HCl (64%). (k) TPAP, 4-ÅMS, MeCN, CH_2_Cl_2_ (40%).

(−)-Phytocassane D was synthesized from the (*R*)-Wieland–Miescher ketone **1** (Scheme 1) and, after a comparison with the spectral data of the authentic natural product, its absolute configuration was confirmed as *ent*-cassane (Table 3, entry 2) [144].

Compound **1** was converted into **2** through a six steps sequence where two methyl groups on C-(4) were inserted. A Robinson annulation of **2** gave the unsaturated tricyclic ketone **3** that was converted into **4** by passing through the saturated tricyclic ketone and inserting the double bond by employing a sulfinate ester. Compound **4** was reacted with Me_2_CuLi to give, after a medium-pressure liquid chromatography, compound **5** in a discrete yield. The latter was converted into **6** using a four-step sequence that consisted of a formylation reaction, a subsequent sodium borohydride reduction, protection of the primary hydroxy group as a tert-butyldiphenylsilyl ether, and finally, a reoxidation of the secondary hydroxy group at C-(12) with PCC. Alkene **7** was obtained with a Shapiro reaction on ketone **6** followed by selective silyl-group deprotection. The double bond in compound **7** was transformed into an epoxy group, while the hydroxyl function was oxidized, yielding an aldehyde that, by treatment with pyrrolidine, yielded the α,β-unsaturated γ-hydroxy aldehyde **8**. The latter was converted into ketone **9** after a four-reaction sequence that included an olefination, an acetylation, the removal of the *tert*-butyldimethylsilyl protecting group, and finally, a pyridinium chlorochromate oxidation. The oxygenated function on C-(2) was inserted by transforming the ketone **9** into the corresponding silyl enol ether, epoxidizing the enol double bond, and treating the epoxide with methanolic oxalic acid. Oxidation of the keto-alcohol with Dess–Martin periodinane gave the corresponding α-diketone, which furnished the enol ether **10** under silylation conditions. Finally, a reduction and a cleavage of the *tert*-butyldimethylsilyl protecting group gave **11,** and selective oxidation of the allylic hydroxy group at C-11 afforded (−)-phytocassane D.

### 4.3. Oryzalexin Chemical Synthesis

**Scheme 2:**
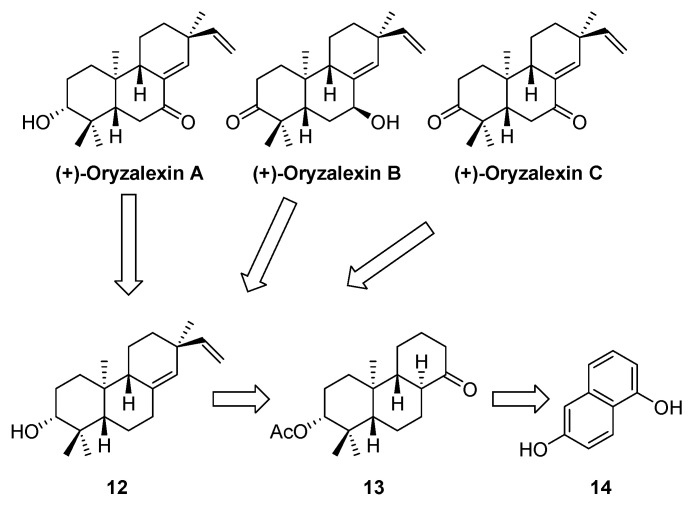
Retrosynthetic scheme for (+)-oryzalexins A–B.

(+)-Oryzalexins A–C were synthesized by Mori and Waku in 1985 [145] (Table 3, entry 3). Their synthesis was planned in a way to have a key intermediate (compound **12** in Scheme 2) that, after allylic oxidation, would provide the target molecule. Compound **12** would be synthesized from tricyclic ketone **13**, whose preparation could be carried out from the commercially available naphthalene derivative **14**.

**Scheme 3:**
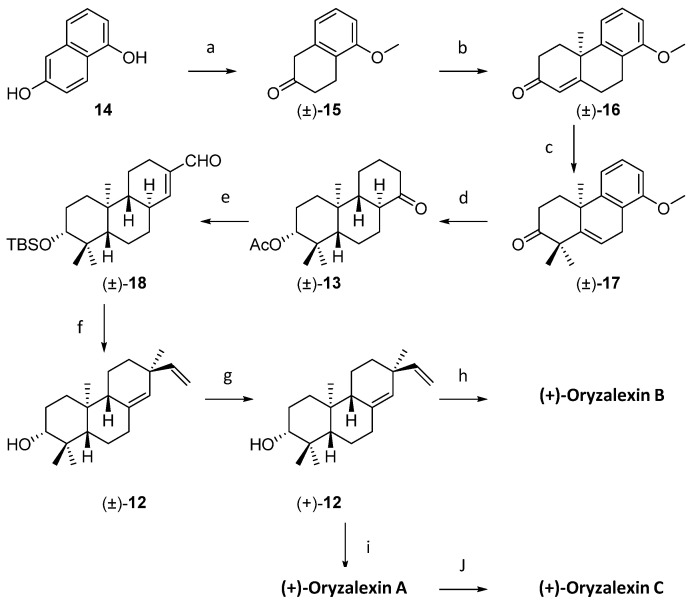
(+)-Oryzalexins A–C chemical synthesis. Reagents and conditions: (a) (i) (CH_3_O)_2_SO_2_, NaOH, Δ; (ii) Na, boiling alcohol (73%, 2 steps). (b) (i) pyrrolidine, C_6_H_6_, Δ; (ii) CH_3_I, 1,4-dioxane, Δ; (iii) (C_2_H_5_)_2_NCH_2_CH_2_COCH_3_, CH_3_I, C_6_H_6_, 0 °C (59% 3 steps). (c) KOC(CH_3_)_3_, CH_3_I (75%). (d) (i) HOCOCH_3_, Pd on C, H_2_; (ii) NaI, CH_3_CN, (CH_3_)_3_SiCl, room temp.; (iii) THF, Li, liquid NH_3_, −60 °C; (iv) TsOH, CH_3_OCOCH_3_, Δ; (v) Raney Ni, H_2_; (vi) Jones CrO_3_ (34% 6 steps). (e) (i) NaOCH_3_, HCOOCH_2_CH_3_, C_6_H_6_; (ii) TsOH, C_6_H_6_, BuSH, Δ; (iii) t-BuMe_2_SiCl, DMF, imidazole, room temp.; NaBH_4_, CH_3_CH_2_OH; (iv) CH_3_CH_2_OH, CdCO_3_, HgCl_2_, Δ (58%, 4 steps). (f) (i) C_6_H_6_, KOC(CH_3_)_3_, CH_3_I, Δ; (ii) Ph_3_P=CH_2_, THF; (iii) HF, CH_3_CN (40%, 3 steps). (g) (i) (−)-camphanoyl chloride, 0 °C, diastereoisomer separation by column chromatography; (ii) conc HCl, CH_3_OH (40%, 2 steps). (h) (i) acetone, Jones CrO_3_; (ii) C_6_H_6_, SeO_2_, HOCOCH_3_, H_2_O (66% 2 steps). (i) (i) CH_3_COOCOCH_3_, pyridine; (ii) C_6_H_6_, SeO_2_, HOCOCH_3_, H_2_O; (iii) (CH_3_)_2_SO, CH_3_COOCOCH_3_, (iv) K_2_CO_3_, CH_3_OH (23%, 4 steps). (j) acetone, Jones CrO_3_ (100%).

Compound **14** was converted, via ketone **15**, into racemic tricyclic enone **16** (Scheme 3). The latter compound was demethylated to give (±)-**17,** which, in a six-step sequence, was transformed into (±)-**13** with the oxygenated group on C-(3) oriented correctly. The C-ring of the ketone (±)-**13** has been elaborated to have the α,β-unsaturated aldehyde on the C-(13) present in compound (±)-**18**. This group is indeed necessary for the insertion of the methyl and the vinyl group on the isopimarane skeleton of the oryzalexins. The key compound **12** was initially synthetized in a racemic fashion, and for its optical resolution, the compound was reacted with different chiral acyl-chloride to give a diastereomeric mixture of esters. Acylation of **12** with (−)-camphanyl chloride yielded better results. Finally, (+)-**12** was converted to oryzalexins A–C by means of allylic oxidation with SeO_2_.

### 4.4. Sakuranetin Chemical Synthesis

**Scheme 4:**
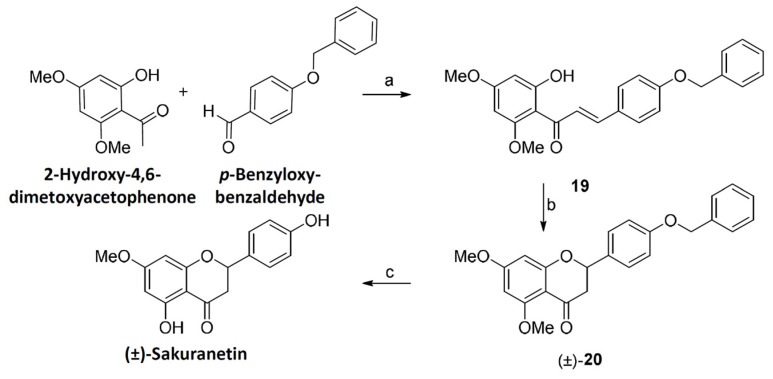
(±)-Sakuranetin chemical synthesis. Reagents and conditions: (a) 2-methoxyethanol (79%). (b) H_3_PO_4_ (40%); (c) (i) H_2_/Pd-C, EtOAc; (ii) BCl_3_, CH_2_Cl_2_ (32% 2 steps).

The first total synthesis of (±)-sakuranetin was performed in 1987 by Mizuno and co-workers [148], and it was made during a study on the structure of a flavanone extracted from the seeds of *Coptis japonica* var. *dissecta* (Table 3, entry 5, Scheme 4).

The synthesis is relatively simple. To give chalcone **19** that, upon acidic treatment, gave the racemic flavanone **20**, 2-hydroxy-4,6-dimethoxyacetophenone was condensed with *p*-benzyloxybenzaldehyde. The latter, after a debenzylation reaction and deprotection of only one of the methoxy groups, gave racemic sakuranetin.

The second total synthesis, always racemic, was undertaken during a study on diinsininolone synthesis (Table 3, entry 5) [151].

**Scheme 5:**
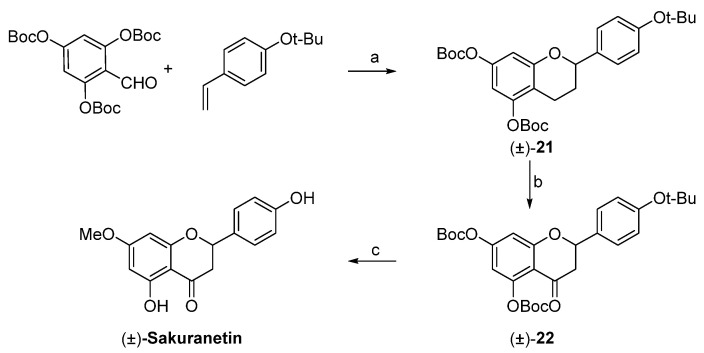
(±)-Sakuranetin chemical synthesis. Reagents and conditions: (a) LiAlH_4_, MgBr_2_ (45%). (b) DDQ (2 equiv), H_2_O, CH_2_Cl_2_ (88%); (c) (i) ZnBr_2_, CH_2_Cl_2_; (ii) K_2_CO_3_, MeI, acetone (36%, 2 steps).

In this synthesis (Scheme 5), the flavanone skeleton was formed through an ortho-quinone methide cycloaddition–oxidation sequence. The ortho-quinone methide was formed in situ, and it reacted in a cycloaddition reaction with *p*-tertbutoxystirene to produce flavan (±)-**21** that is converted into sakuranetin via (±)-**22**.

In addition to these two total syntheses, there are a series of articles in the literature (Table 2, entries 4 and 5) relating to the semisynthesis of sakuranetin by methylation of the natural product naringenin [146,147,149,150,152,153,154,155].

## 5. Biological Activities of Rice Phytoalexins

### 5.1. Bioactivities of Diterpenoid Phytoalexins

Rice phytoalexins have been investigated mainly for their antimicrobial activity, which is linked to their role in plant–pathogen interactions, and for their inhibitory activity on seed germination and plant development, which is instead correlated with their role in plant–plant allelopathic interactions. In recent years, a growing number of studies have revealed a wide variety of biological activities (Table 4) and possible applications of these biomolecules, especially in the pharmaceutical field.

#### 5.1.1. Momilactones

As previously mentioned, momilactones are secondary metabolites belonging to the (9β-H)-pimarane diterpene family, found not only in cultivated and wild rice [27,28,44,127] but also in other Poaceae such as *Echinochloa crus-galli* (barnyard grass) [9], as well as in the mosses *Calohypnum plumiforme* [48,62], and *Plagiomnium acutum* [47]. Several biological activities have been attributed to momilactones (Table 4), some of which are directly related to their biological role in plant-pathogen and plant–plant interactions. Momilactones also exhibit pharmacological activities, which make them potential candidates for the development of novel drugs, cosmetics, and additives for health-promoting foods.

- Antimicrobial and allelopathic activity:

*Magnaporthe grisea*, the causal agent of rice blast disease, is a major devastating pathogen resulting in a loss of 40% of global yield [191]. This ascomycete can infect more than 130 Poaceae species, including barley, wheat, and millet [192,193]. The anti-blast activity of momilactones A and B was first reported in 1977 by Cartwright et al. [56]. Following this discovery, several other metabolites isolated from resistant rice strains were tested against this fungal pathogen. Among them, momilactone B exhibited the highest power against both spore germination and germ tube growth of *M. grisea* [28]. The superior antifungal activity of this compound was then confirmed by tests carried out on different fungal pathogens, including *Botrytis cinerea*, *Fusarium solani*, and *Colletrotrichum gloeosporioides* [19]. In addition, momilactone B exhibited significantly higher antibacterial activity than momilactone A against different bacteria such as *Pseudomonas ovalis*, *Bacillus cereus*, and *B. pumilus* [19].

The allelopathic properties of momilactones A and B were soon recognized [27]. It has been observed that, when co-cultivated with rice, the growth of other plant species like barnyard grass [164,165,166,169] and *Alisma plantago-aquatica* (the common water plantain) [167], two of the most disruptive rice weeds, was inhibited. Similar results were obtained using model plants such as *Medicago sativa* (alfalfa) and *Lactuca sativa* (lettuce) [55,157,158].

Momilactone B inhibited the growth of cress (*Lepidium sativum* L.) and lettuce (*Lactuca sativa* L.) seedlings at concentrations above 3 and 30 μM, respectively [58]. Momilactones A and B exhibited strong herbicidal activity against duckweed (*Lemna paucicostata* Hegelm 381) [160] and quantitatively inhibited the germination and growth of three weed species (*Amaranthus retroflexus* L., *Cyperus difformis* L., and *Leptochloa chinensis* L.) at concentrations ranging from 4 to 20 ppm [160]. Further studies confirmed that momilactones A and B accumulate in the roots of rice seedlings and can be released into the environment as root exudates [60,159,194,195]. Expression analyses of diterpene cyclase genes involved in the biosynthesis of momilactones and phytocassanes suggest that rice roots are not only responsible for the accumulation and exudation of these metabolites but also for their production [50]. The role of momilactones as allelochemicals was confirmed via reverse genetics, using knock-outs of relevant diterpene synthase genes (*OsCPS4* and *OsKSL4*, see Section 3.1 and Figure 6 and Figure 7) [190].

An interesting study by Kato-Noguchi and Ino [169] showed that rice can perceive some chemicals released into the environment by barnyard grass. Rice plants respond to the presence of this weed by producing and secreting momilactone B into the surrounding environment. On the other hand, this metabolite induces allelochemical activity in barnyard grass. This suggests that during their evolution, rice and barnyard grass may have developed chemical crosstalk to promote the defense mechanisms against biotic stress conditions by detecting certain key compounds [169].

The group of Kato-Noguchi and collaborators investigated the mode of action of momilactones A and B using the model plant *Arabidopsis thaliana* [196,197,198]. They first observed that momilactones were absorbed by *A. thaliana* in proportion to their exogenous levels and that their inhibitory effects on root and hypocotyl growth were related to their endogenous levels [197]. They then investigated protein expression in the same model plant in response to momilactone treatment. In Arabidopsis plants treated with momilactones A and B, it was observed a higher amount of cruciferina, cruciferin 2, and cruciferin 3 compared to the control. The breakdown of cruciferins and cruciferina is indeed essential for seedling growth as it provides the initial source of nitrogen for seed germination. These results suggest that momilactones may inhibit the germination of Arabidopsis seeds by inhibiting the degradation of these proteins.

Further insights into the role of momilactones as allelochemicals can be found in Table 4 in an excellent review recently published by Serra Serra et al. [12].

- Anti-cancer activity:

In 2005, Chung et al. [160] evaluated the cytotoxic activity of seven compounds isolated from rice hulls. Three of these, namely orizaterpenol and momilactones A and B, showed cytotoxic effects against murine P388 leukemia cells. Momilactone B was found to be significantly more active than momilactone A and orizaterpenol (IC_50_ 0.07, 0.85, and 4.2, respectively) [171]. In 2007 Kim and colleagues [172] evaluated the cytotoxic activity of momilactone B on human colon cancer HT-29 and SW620 cells, which exhibited strong tolerance to anticancer agents in vitro and in vivo in previous studies. Through MTT-dye reduction, lactate dehydrogenase (LDH), and colony-forming ability assays, they highlighted the potential of momilactone B as a novel therapeutic agent to induce cell death in human colon cancer cells [172].

In a study by Lee and colleagues [174], the anticancer activity of momilactone B was demonstrated in blood cancer cells, including human HL-60 leukemia cells, Jurkat human leukemic T cells, rat basophilic leukemia RBL-2H3 cells, and p815 mouse mastocytoma P-815 cells, at concentrations below 6 mM. The cytotoxic effect of momilactone B on Jurkat cells was associated with its apoptosis-inducing activity via caspases and mitochondria.

- Other activities:

Recently, Quan and collaborators [182,183] investigated the anti-diabetic and anti-obesity activity of momilactones A and B. By in vitro assays, they showed potent inhibitory activity of momilactones on key enzymes related to diabetes. The inhibition of pancreatic α-amylase and α-glucosidase was significantly higher than the known diabetes inhibitor γ-oryzanol. In addition, a strong anti-trypsin activity was recorded [183].

In 2016, Xuan and co-workers [177] investigated the contents of momilactones in 30 rice cultivars of different origins, including hybrid, foreign, local, sticky, upland sticky, and upland rice of the two subspecies Japonica and Indica. They found that momilactones in rice are more related to salinity and drought tolerance than weed resistance. The correlation between momilactone A and B content and weed resistance was very low, with r^2^ coefficients of 0.001 and 0.09, while the correlation with drought tolerance was much higher, with r^2^ of 0.65 and 0.27, respectively [177].

In 2019 Quan and colleagues [184] investigated the antioxidant and anti-skin-aging activities of momilactones A and B in comparison with tricin, a well-known antioxidant and antiaging rice flavonoid. ABTS assay and in vitro enzymatic assays on pancreatic tyrosinase and elastase highlighted the synergistic activity of momilactones A and B, whose mixture showed significantly greater activity than single momilactones and tricin [184].

#### 5.1.2. Oryzalexins

As mentioned above, oryzalexins A–F are distinguished from oryzalexin S both by both the biosynthetic pathway and their molecular structure (Figure 3 and Figure 6).

Contrary to what has been reported for momilactones, the literature concerning oryzalexins’ biological activity is surprisingly limited. The most widely known bioactivity associated with these diterpenoid compounds is the antimicrobic activity against *M. grisea*, the rice blast fungus [20,187]. Although many reviews from the last decade report this activity [1,199,200,201,202], they typically refer to literature from the late 1900s that merely scratched the surface on this topic, focusing on *M. grisea* spore inhibition [33,185,186].

Interestingly, recent reports suggest that oryzalexins may have other potentially valuable capabilities. Cho and colleagues [43] recently reported a potential anti-inflammatory activity of oryzalexin A, which has been shown to possess an inhibitory activity on NO production by mouse macrophage RAW264.7 cells. Furthermore, Jain and Das [188] observed that oryzalexin B, in combination with other natural compounds, seems to be able to bind six potential receptors in estrogen receptor-positive breast cancer, suggesting another potential use in medicine.

Oryzalexin S also shows a mild allelopathic effect in lettuce and barnyard grass [190] and, along with other oryzalexins, seems to be able to affect stomatal closure, playing a role in drought resistance [189].

Regardless of their role, oryzalexins have been shown to accumulate after exposure to fungal proteins [203] and oligosaccharides [204,205,206,207], as well as fungal [208] and nematode [209] infection. Abiotic factors also seem to induce the accumulation of oryzalexins, such as heavy metal ions [54], salicylic acid [210], and UV radiation [53,211].

#### 5.1.3. Phytocassanes

Despite their known role as phytoalexins, literature regarding phytocassanes and their biological activity is surprisingly scarce. In the last decade of the 20th century, known members of this class of diterpenes (i.e., phytocassanes A–E) were found to effectively inhibit spore germination when rice plants were infected with the rice blast fungus *M. grisea*, the rice sheath blight fungus *Rhizoctonia solani*, and the pathogenic potato fungus *Phytophthora infestans* [29,30]. Over the last decade, Horie and co-workers [31] confirmed these observations, including the recently discovered phytocassane F in their tests. During this last work, an increase in the production of phytocassanes after exposure of rice leaves to UV light was also observed [31], providing insights into the role of these compounds in response to abiotic stress. Considering biotic factors, increases in phytocassane production have been observed after exposure to fungal inoculation [212], *Tricoderma viride*-derived xylanase [213,214], cerebrosides A, B, and C [215,216], cholic acid [217,218], and mannan oligosaccharides [219], demonstrating their implication in response to a wide array of biological challenges. More recently, phytocassanes have been confirmed to play an active role in plant response to stress in general. Knock-out lines with deletion of biosynthetic gene clusters from chromosome 2, associated with phytocassane biosynthesis, were shown to be more susceptible to fungal blast and bacterial leaf blight than lines with deleted biosynthetic gene clusters from chromosome 4, associated with momilactone biosynthesis. These mutants also exhibited a drought and temperature-sensitive phenotype [126].

### 5.2. Bioactivities of Phenolic Phytoalexins

Sakuranetin is the main phenolic phytoalexin in rice. It showed remarkable antifungal activity against phytopathogenic fungi, including *M. grisea* and *R. solani* [70,92,93]. In addition to the antifungal activity, this flavanone exhibits a wide range of other biological activities that makes it attractive to the pharmaceutical industry. Some of the major sakuranetin bioactivities are listed in Table 5.

In a recent paper, Moulishankar and Lakshmanan [228] investigated the 3D and 2D interactions between 26 naturally occurring flavonoids and 11 target enzymes through molecular docking (a key tool used in structural molecular biology and computer-assisted drug design). They found that sakuranetin binds to several targets related to specific bioactivities, namely 4KIK (anticancer activity by IkB kinase inhibition), 4HZ5 (antibacterial activity by DNA gyrase B and topoisomerase IV inhibition), and 3LN0 (anti-inflammatory activity by cyclo-oxygenase inhibition). Further studies on the interaction between sakuranetin and specific targets involved in human diseases will contribute to the elucidation of molecular mechanisms underlying the bioactivities of this compound, which are still unknown or not fully understood.

According to Miyazawa and colleagues [220], sakuranetin suppresses *umu* gene expression during the SOS response against AF-2 in *Salmonella typhimurium*. The SOS response is thought to be triggered by an alteration in DNA synthesis, either directly by DNA damage that blocks the replication fork or indirectly by antibiotics (e.g., novobiocin) that inhibit DNA synthesis. The *umu* assay was developed to evaluate the genotoxic effects of environmental mutagens and carcinogens by examining the expression of a gene from the SOS family to detect DNA-damaging agents.

Sakuranetin has been shown to inhibit cancer growth both in vitro and in vivo. The induction of cell death by apoptosis appears to be the main mechanism involved in this bioactivity. As shown by Park et al. [18], sakuranetin inhibits the proliferation of human colon cancer HCT-116 cells with an IC_50_ value of approximately 68.8 μg/mL. According to Drira and Sakamoto [222], sakuranetin strongly promotes melanogenesis in murine B16BL6 melanoma cells by inhibiting ERK1/2 and PI3K/AKT signaling pathways, leading to increased expression of the Tyr family genes *TRP1* and *TRP2*. Additionally, they found that sakuranetin reduced the proliferation rate of melanoma cells at concentrations ≥15 µmol/L without directly affecting cell viability. Based on these findings, sakuranetin appears to be a promising candidate for anticancer drug development.

In a study aimed at identifying antiallergic compounds in resin extracts of *Xanthorrhoea hastilis* R. BR. (Xanthorrhoeaceae), Ogawa and co-workers [85] isolated three chalcones and six flavanones, including sakuranetin, through bioassay-directed fractionation. In vivo assays and measurements of platelet aggregation demonstrated that sakuranetin is one of the active ingredients responsible for the antiallergic activity of *X. hastilis* extracts.

Between 1999 and 2005, several studies highlighted the presence of sesquiterpenes and flavonoids with anti-inflammatory activity in *Inula viscosa* (L.) Aiton (Asteraceae), an herbaceous plant known for its effectiveness against skin inflammations [223,224,225,226,227,228,229,230]. In 2007 Hernández and colleagues [223] tested the anti-inflammatory properties of three flavanones isolated from *I. viscosa*, namely 7-O-methylaromadendrin, 3-acetyl-7-*O*-methylaromadendrin, and sakuranetin. Sakuranetin was the most active in vitro, inhibiting the production of LTB4, acting directly on the 5-LOX enzyme and regulating secretory processes such as elastase release. Although the anti-inflammatory activity of flavonoids is usually related to their antioxidant activity, the results of Hernández et al. [223] suggest a possible non-redox inhibition of lipoxygenases, as well as a blockage of some proteins implicated in exocytotic mechanisms.

Saito and colleagues [224] observed that, even in the absence of adipogenic hormonal stimuli, sakuranetin strongly promoted both the differentiation of 3T3-L1 preadipocytes into adipocytes and the expression of genes associated with the development of adipocyte phenotypes. They also observed that glucose uptake in differentiated 3T3-L1 fat cells was stimulated by sakuranetin, suggesting that it may contribute to the maintenance of glucose homeostasis in animals.

Sakoda and co-workers [225] evaluated the impact of sakuranetin on vascular and lung parenchyma alterations in a murine model of chronic allergic pulmonary inflammation. In most of the parameters evaluated by histopathological analysis (Table 5), the effects of sakuranetin were similar to those of the steroidal anti-inflammatory drug dexamethasone. The authors speculated that the reduction in the number of eosinophils and elastic fibers in pulmonary vessels and lung parenchyma, promoted by sakuranetin, results from the reduction of oxidative stress and the levels of transcription factors NF-kB and VEGF in the lung.

Zhang and collaborators [226] identified sakuranetin as a new inhibitor of the carrier protein β-hydroxyacyl-acyl dehydratase from *Helicobacter pylori* (HpFabZ). Sakuranetin was compared with two other flavonoids that had already been reported as HpFabZ inhibitors, namely quercetin and apigenin [226]. Sakuranetin exhibited significantly greater inhibitory activity than the other compounds (IC**_50_** in μM: 2.0, 39.3, and 11.0, respectively). Complex crystal structure analysis in combination with kinetic enzyme assays indicated that the compounds of interest act as competitive inhibitors of HpFabZ by binding to the B tunnel entrance of the substrate or by plugging into the C tunnel near the catalytic residues mainly through hydrophobic interactions and hydrogen-bond pattern.

Grecco and colleagues [227] studied the activity of sakuranetin against promastigotes and amastigotes of *Leishmania* spp. and trypomastigotes and amastigotes of *Trypanosoma cruzi*. Sakuranetin was found to be active against *L. amazonensis*, *L. braziliensis*, *L. major*, and *L. chagasi* (with IC_50_ between 43 and 52 μg/mL) and against *T. cruzi* trypomastigotes (IC_50_ = 20.17 μg/mL). Interestingly, sakuranetin was methylated to produce sakuranetin-4′-methyl ether, which was found to be inactive against both *Leishmania* spp. and *T. cruzi*, suggesting that the presence of a hydroxyl group at C-4′ together with a methoxyl group at C-7 is required for the antiparasitic activity. Further drug design studies targeting sakuranetin derivatives could contribute to the development of promising therapeutic agents for leishmaniasis and Chagas’ disease.

In a study conducted by Park and co-workers [74], phenolic rice phytoalexins were evaluated for their antimicrobial activity against phytopathogenic fungi and bacteria. Inhibition of *Bipolaris oryzae* (rice brown spot fungus) growth was observed with N-*trans*-cinnamoyltryptamine. In addition to *B. oryzae*, sakuranetin was active against *Magnaporthe grisea* (rice blast fungus) and *Rhizoctonia solani* (rice sheath blight fungus). Phenylamides (N-*trans*-cinnamoyltryptamine and N-*p*-coumaroylserotonin) and sakuranetin induced by UV exposure showed antibacterial activity against rice pathogens for blight (*Xanthomonas oryzae* pv. *oryzae*), grain rot (*Burkholderia glumae*), and leaf streak (*X. oryzae* pv. *oryzicola*) diseases.

## 6. Conclusions

With this review, we intend to provide an overview of the literature produced over the last few decades on the distribution, biosynthesis, chemical synthesis, and biological activity of rice phytoalexins.

Rice has been shown to biosynthesize quite a wide array of phytoalexins, mostly in the form of diterpenoids (such as momilactones, phytocassanes, and oryzalexins) but also as phenolic compounds (such as sakuranetin and phenylamides). The current knowledge on rice phytoalexins is very large and constantly growing; therefore, this review cannot claim to be exhaustive on the subject.

As emerges from this review, in recent decades, considerable attention has been paid by the scientific community to the study of the pathways involved in the biosynthesis of rice phytoalexins. Conversely, studies on rice phytoalexin bioactivity mainly focused on their antifungal activity, primarily against the rice blast fungus *Magnaporthe grisea*. The results of the few studies conducted to date on other microorganisms are very encouraging, suggesting that the spectrum of action of these compounds could be much broader than we now know today.

Interestingly, some diterpenoids, such as momilactones, have been shown to play different roles, being involved both in the defense of the plant against pathogens (phytoalexins) and in plant–plant allelopathic interactions (allelochemicals). These secondary metabolites may thus reveal novel and interesting functions. It would, therefore, be desirable, in the near future, to further intensify investigations on the bioactivity of rice phytoalexins to better understand both their multiple functions in the plant and their potential applications, mainly in the biomedical field.

Great efforts have been made in the past to select rice varieties that best meet the needs and demands of farmers, the processing industry, and consumers. As emerges from the research reported in this review, many of the most valuable rice varieties have low levels of metabolites involved in defense against biotic stresses (e.g., phytoanticipins, phytoalexins, allelochemicals). In recent years, genetic improvement programs have focused on the selection of varieties with greater natural defenses and a reduced need for synthetic pesticides and herbicides, which pose a risk to humans and the environment. In the near future, a major challenge will be to obtain new varieties that have a favorable balance between natural defenses, productivity, and organoleptic and nutritional characteristics.

## Figures and Tables

**Table 3:** Rice phytoalexin chemical synthesis.

Entry	Compound	Skeletal Type	References
1	(±)-Momilactone A	(9β-H)-Pimarane	[143]
2	(−)-Phytocassane D	*ent*-Cassane	[144]
3	(+)-Oryzalexin A, B and C	*ent*-Isopimarane	[145]
4	(−)-Sakuranetin	Flavanone	[146,147] *,
5	(±)-Sakuranetin	Flavanone	[148], [149,150] *, [151], [152,153,154,155] *

* Semisynthesis of sakuranetin from naringenin.

**Table 4:** Biological activities of rice diterpenoid phytoalexins.

Metabolite/s	Activity	Experimental Model	References
Momilactones	Antimicrobial and allelopathic	Structural characterization of momilactones A and B isolated from rice husk (cv. Koshihikari), which inhibited rice root growth at less than 100 ppm.	[27]
		Evaluation of inhibitory activity of momilactones A and B isolated from rice (cvs. Koshihikari and Surjamukhi) seed hulls on rice and lettuce seed germination.	[55]
		Structural characterization of momilactone C isolated from rice husk (cv. Koshihikari) and evaluation of inhibitory activity toward germination of lettuce seeds.	[42]
		Increased responsiveness of rice to the infection by *M. grisea* after treatment of leaves with the resistance inducer WL28325 (2,2-dichloro-3,3-dimethylcyclopropane carboxylic acid) in terms of momilactones A and B synthesis.	[56]
		Evaluation of inhibitory activities of momilactones A and B isolated from rice (cv. Koshihikari) on lettuce seed germination and rice root growth. Bioassays with several semisynthetic momilactone derivatives to establish the functional groups responsible for the bioactivity.	[157]
		Structural characterization of momilactones A and B from coleoptiles of etiolated plantlets of rice (cv. Sasashigure) exposed to UV radiation. Evaluation of anti-fungal activity on *Cladosporium cucumerinum* through TLC bioassay.	[28]
		Evaluation of inhibitory effect of rice root exudates (cvs. Hinohikari, Nipponbare, Norin 8, Kamenoo, Kinuhikari, Koshihikari, Sasanishiki, and Yukihikari) on co-cultivated seedlings of alfalfa (*Medicago sativa* L.), cress (*Lepidium sativum* L.) or lettuce (*Lactuca sativa* L.).	[158]
		Evaluation of inhibitory activity of momilactone B in rice (cv. Koshihikari) root exudates on growth of cress and lettuce.	[58,159]
		Evaluation of phytotoxicity of momilactones A and B and other putative allelochemicals from rice (cv. Hochokjindo) hulls against duckweed (*Lemna paucicostata*). Evaluation of inhibitory effects of identified compounds on germination and growth of the three weed species *Amaranthus retroflexus*, *Cyperus difformis*, and *Leptochloa chinensis*.	[160]
		Release of momilactone B and other allelochemicals into the soil promoted by the presence of barnyard grass by two allelopathic rice varieties PI312777 [from USDA-ARS rice germplasm collection [161] and Huagan-1, and the non-allelopathic rice variety Huajingxian.	[162]
		Evaluation of allelopathic potential of rice hull extracts from ninety-nine rice varieties containing momilactones A and B on barnyard grass germination and growth.	[163]
		Evaluation of inhibitory activity of acetone extracts from the moss *Calohypnum plumiforme* (syn. *Hypnum plumaeforme*) on the growth of angiosperms (*Arabidopsis thaliana*, *Nicotiana tabacum*), mosses (*Physcomitrella patens* and *H. plumaeforme*), and a liverwort (*Jungermannia subulata* cultured cells).	[48]
		Evaluation of herbicidal activity (inhibition of germination and elongation of shoot and root) of momilactones A and B from rice hulls on barnyard grass and monochoria (*Monochoria vaginalis*). Evaluation of antifungal activity by agar dilution method against *Botrytis cinerea*, *Fusarium solani*, *F. oxysporum*, and *Colletrotrichum gloeosporioides.* Evaluation of antibacterial activity by disc diffusion method against *Pseudomonus ovalis*, *Bacillus cereus*, *B. pumilus*, and *Escherichia coli*.	[19]
		Evaluation of growth inhibitory activity (lengths of hypocotyls) of momilactones A and B released in the medium by in vitro-grown rice (cv. Nipponbare) seedlings on lettuce and Chinese cabbage (*Brassica rapa*).	[50]
		Evaluation of inhibitory activity of momilactone A from root exudates of rice (cv. Koshihikari) on growth of cress (length of root and hypocotyl).	[60,61]
		Elicitation of momilactone accumulation and secretion in *C. plumiforme* through UV, jasmonic acid, metals (CuCl_2_ and FeCl_2_), and a protein phosphatase inhibitor (cantharidin).	[63,64]
		Evaluation of inhibitory activity of H_2_O:MeOH extracts of soil under colonies of *C. plumiforme* on growth of cress (*Lepidium sativum*), lettuce (*Lactuca sativa*), lucerne (*Medicago sativa*), ryegrass (*Lolium multiflorum*), timothy (*Phleum pratense*), *Digitaria sanguinalis*, and *Echinochloa crus-galli*.	[65]
		Evaluation of inhibitory activity of momilactones A and B from root exudates of rice (cv. Koshihikari) on growth of barnyard grass (length of shoot and hypocotyl). Evaluation of allelopathic activity of six rice cvs. (Hinohikari, Kamenoo, Kinuhikari, Koshihikari, Nipponbare, Norin 8, Sasanishiki, and Yukihikari) by donor–receiver bioassay.	[164]
		Evaluation of momilactone B production and release in rice (*O. sativa*) and barnyard grass (*E. crus-galli*) co-cultures.	[165,166]
		Screening of 41 different rice cvs. for their ability to reduce germination, root growth, and root dry weight of *Alisma plantago-aquatica*. Quantification of momilactone B in the studied rice cvs.	[167]
		Evaluation of allelopathic activities of momilactones A and B from husks of rice (cv. Koshihikari) against different plant species, including nine weed species [Cress, lettuce, alfalfa (*Medicago sativa*), ryegrass (*Lolium multiflorum*), timothy, barnyard grass, *Echinochloa colonum*, crabgrass (*Digitaria sanguinalis*), and *Arabidopsis thaliana*], and four rice cvs. (Koshihikari, Nipponbare, Norin 8, and Sasanishiki).	[168]
		Evaluation of allelopathic activity of barnyard grass in response to momilactone B from rice (cv. Koshihikari) seedlings or root exudates.	[169]
		Evaluation of inhibitory activity of momilactones A, B, E from rice (cv. Koshihikari) husk on germination rate and root and shoot elongation of lettuce, barnyard grass, and tall goldenrod (*Solidago altissima*).	[170]
	Anti-cancer	Evaluation of cytotoxic activity of momilactones A and B from rice (cv. Hochokjindo) hulls against P388 murine leukemia cells.	[171]
		Evaluation of cytotoxic and antitumor activity of methanolic extract and momilactone B from rice hulls by MTT-dye reduction assay against human colon cancer cells and colonic aberrant crypt foci (ACF) assay in 1,2-dimethylhydrazine (DMH)-injected F344 male rats, respectively.	[172]
		Investigation of the molecular mechanism responsible for the inhibitory effects of momilactone B on the growth of cultured human breast cancer T47D cells.	[173]
		Evaluation of antitumor efficacy by inducing apoptosis in several mammalian blood cancer cells, including human leukemic T cells.	[174]
		Investigation of the molecular mechanism responsible for the inhibitory effects of momilactone B on cultured human leukemia U937 cell growth.	[175]
		Investigation of the molecular mechanism responsible for the cytotoxic activity of momilactones A and B against multiple myeloma U266 and acute promyelocytic leukemia HL-60 cell lines.	[176]
	Increased tolerance to salinity and drought stresses	Correlation between the content of momilactones A and B and tolerance to salinity and drought in 30 rice cultivars.	[177]
		Induction of momilactones A and B by application of exogenous vanillic acid and *p*-hydroxybenzoic acid to two rice cvs., one drought-tolerant (Nep nanh ngua Hai phong), and one drought-susceptible rice (Re nuoc).	[178]
		Enhancement of momilactone B and phenolic acids production by exogenous application of MgSO_4_ to salinity tolerant (BC15) and salinity susceptible (DT84DB) rice varieties [179].	[180]
	Increased tolerance to UV and chilling stresses	Induction of expression of genes related to the biosynthesis of momilactones and phenolics and enhancement of their accumulation by UV.	[181]
	Anti-diabetic and anti-obesity	Evaluation of in vitro inhibitory activity of momilactones A and B from rice (cv. Koshihikari) hulls on α-amylase and α-glucosidase (in comparison with the known commercial diabetes inhibitors, acarbose, and quercetin).	[182]
		Evaluation of in vitro inhibitory activity of momilactones A and B from rice bran on pancreatic α-amylase and α-glucosidase (in comparison with the known diabetes inhibitor γ-oryzanol).	[183]
	Antioxidant	Evaluation of antioxidant activity of momilactones A and B from rice hulls through DPPH radical scavenging capacity assay.	[19]
		Evaluation of antioxidant activity of momilactones A and B from rice grains of different cultivars (Koshihikari, Shinnosuke, Seiten no hekireki, Ginga no shizuku, Ho no mai) through ABTS radical cation decolorization assay.	[184]
	Anti-inflammatory	Structural characterization of momilactones D and E from rice (cv. Chucheongbyeo) root and evaluation of their ability to inhibit the production of NO and iNOS mRNA and protein expression in LPS-stimulated RAW264.7 macrophages.	[43]
	Anti-aging	Evaluation of the ability of momilactones A and B from rice grains of different cultivars (Koshihikari, Shinnosuke, Seiten no hekireki, Ginga no shizuku, Ho no mai) to relieve wrinkles, skin, and freckles by in vitro enzymatic assays on pancreatic elastase and tyrosinase.	[184]
Oryzalexins A-F	Antimicrobic	Evaluation of inhibitory activity of oryzalexins A, B, and C against *M. grisea.*	[33]
		Evaluation of inhibitory activity of oryzalexin D against *M. grisea.*	[185]
		Evaluation of inhibitory activity of oryzalexin A, B, and C synthetic enantiomers against *M. grisea.*	[186]
		Evaluation of inhibitory activity of oryzalexins A-F against *Magnaporthe grisea.*	[20]
		Evaluation of inhibitory activity of oryzalexin B and sakuranetin induced in rice leaves by elicitation with fungal metabolites and nanoparticles on spore production in *M. grisea.*	[187]
	Anti-cancer	Evaluation of binding potential of a combination of oryzalexin B and other compounds on six potential receptors in estrogen receptor-positive breast cancer.	[188]
	Regulation of stomatal closure	Evaluation of stomatal closure and susceptibility to drought in *cps2* and *cps4* knockout lines.	[189]
Oryzalexin S	Allelopathic	Evaluation of allelopathic activity of oryzalexin S against *L. sativa* cv. Black-seeded Simpson and *E. crus-galli* cv. Kudiraivali in *O. sativa* Oskls4 knockouts.	[190]
	Antimicrobic	Evaluation of inhibitory activity of oryzalexins S against *M. grisea*.	[20]
	Regulation of stomatal closure	Evaluation of stomatal closure and susceptibility to drought in *cps2* and *cps4* knockout lines.	[189]
Phytocassanes	Antimicrobial	Evaluation of inhibition of *M. grisea* spore germination by phytocassanes A-D from rice leaves infected with *M. grisea* and from rice stems infected with *Rhizoctonia solani.*	[29]
		Evaluation of inhibition of *M. grisea* spore germination by phytocassanes E from rice suspension-cultured cells treated with a mycelial extract of the pathogenic potato fungus *Phytophthora infestans.*	[30]
		Evaluation of inhibition of *M. grisea* spore production by phytocassanes A-F from rice leaves elicited by UV light.	[31]

**Table 5:** Biological activities of sakuranetin.

Biological Activity	Experimental Model	References
Antifungal	*Magnaporthe grisea* (syn. *M. oryzae*, *Pyricularia grisea/oryzae*)—Inhibition of spore germination and germ tube growth.	[70]
	*M. grisea*—Fungal colony growth inhibition assay.	[95]
Antimutagenic	*Salmonella typhimurium* TA1535/pSK1002—Suppressive effect on *umu* gene expression of SOS response against the mutagen AF-2.	[220]
Anticancer	Multidrug-resistant Colo 320 human colon cancer cells—Growth inhibition by induction of apoptosis.	[221]
	Colon carcinoma HCT-116 human cells—Cytotoxicity test through MTT assay.	[18]
	B16BL6 mouse melanoma cells—Dose-dependent stimulation of melanogenesis.	[222]
Antiallergic	Blood flow decrease in the tail vein microcirculation of mice subjected to HEL-sensitization as a monitor—Measurement of platelet aggregation of whole blood induced by ADP through WBA analyzer.	[85]
Anti-inflammatory	In vivo induction of acute inflammation by topical application of TPA to mouse ears or by subcutaneous injection of PLA2 into mouse paws. In vitro tests based on the effect of sakuranetin and other dihydroflavonols on arachidonic acid metabolism and release and/or activity of enzymes implicated in the inflammatory response like elastase, MPO, and PKC.	[223]
Adipogenesis induction—glucose uptake stimulation	Differentiation of 3T3-L1 murine preadipocytes to adipocytes. Expression of genes involved in development of adipocyte phenotypes. Evaluation of basal- and insulin-stimulated glucose uptake.	[224]
Anti-inflammatory and antioxidant	Murine model (male BALB/c mice) of chronic allergic pulmonary inflammation—Histopathological analysis: evaluation of extracellular matrix remodeling, inflammation, and oxidative stress in pulmonary vessels and lung parenchyma; quantification of the vascular wall thickness and the VEGF levels.	[225]
Anti-*Helicobacter pylori*	Study of the interaction between sakuranetin and other flavonoids with HpFabZ protein from *H. pylori* by enzymatic and crystalline structure analyses.	[226]
Antileishmanial and antitrypanosomal	In vitro antileishmanial and antitrypanosomal tests.	[227]

ADP: Adenosine diphosphate; AF-2: Furylfuramide; EC50: Half maximal effective concentration; HEL: Hen egg-white lysozyme; HpFabZ: β-hydroxyacyl-acyl carrier protein dehydratase from *Helicobacter pylori*; MPO: Myeloperoxidase; MTT: Thiazolyl blue tetrazolium bromide; PKC: Protein kinase C; PLA2: Phospholipase A2; TPA: 12-O-Tetradecanoylphorbol-13-acetate; VEGF: Vascular endothelial growth factor; WBA: Whole blood aggregometer.

## Data Availability

Not applicable.

## Abbreviations

3LN0	Structure of compound 5c-S bound at the active site of COX-2
4HZ5	Pyrrolopyrimidine inhibitors of DNA gyrase B and topoisomerase iv
4KIK	Human IkB kinase beta
5-LOX	5-Lipoxygenase
ACC	Acetyl-CoA carboxylase
ADP	Adenosine diphosphate
AF-2	Furylfuramide
BenSer	*N*-Benzoylserotonin
BenTry	*N*-Benzoyltryptamine
BenTyr	*N*-Benzoyltyramine
BGC	Biosynthetic gene cluster
CDP	Copalyl diphosphate
CHI	Chalcone isomerase
CHS	Chalcone synthase
CinSer	*N-trans*-Cinnamoylserotonin
Cin-Try	*N-trans*-Cinnamoyltryptamine
Cin-Tyr	*N-trans*-Cinnamoyltyramine
CM	Chorismate mutase
CouSer	*p*-Coumaroylserotonin
CS	Chorismate synthase
cv.	Cultivar
CYPs	Cytochrome P450 enzymes
DAHP	3-Deoxy-D-arabinoheptulosonate 7-phosphate
DAHPS	3-Deoxy-D-arabinoheptulosonate 7-phosphate synthase
DHQDT/SDH	3-Dehydroquinate dehydratase/shikimate dehydrogenase
DHQS	3-Dehydroquinate synthase
diTPS	Diterpene-synthase
DMAPP	Dimethylallyl diphosphate
EC_50_	Half maximal effective concentration
*ent*-CPP	(*5R*,*9S*,*10S*)-Copalyl diphosphate
ER	Endoplasmic reticulum
FerAgm	*N*-Feruloylagmatine
FerPut	*N*-Feruloylputrescine
FerSer	*N*-Feruloylserotonin
FerTry	*N*-Feruloyltryptamine
FPP	Farnesyl diphosphate
GAs	Gibberellins
GFP	Green fluorescent protein
GGPP	Geranylgeranyl diphosphate
GGPPS	Geranylgeranyl diphosphate synthases
GPP	Geranyl diphosphate
HDR	4-Hydroxy-3-methylbut-2-enyl diphosphate reductase
HEL	Hen egg-white lysozyme
HMBPP	4-Hydroxy-3-methylbut-2-enyl diphosphate
IC50	Half maximal inhibitory concentration
IDS	Isoprenyl diphosphate synthase
IPP	Isopentenyl diphosphate
IPPI	Isopentenyl diphosphate isomerase
IRRI	International Rice Research Institute
JA	Jasmonic acid
LPS	Lipopolysaccharides
LTB4	Leukotriene B4
MEP	Methylerythritol 4-phosphate
MPO	Myeloperoxidase
MTT	3-(4,5-Dimethylthiazol-2-yl)-419 2,5-diphenyltetrazolium bromide
MVA	Mevalonate
NO	Nitric oxide
NOMT	Naringenin 7-O-methyltransferase
OMT	Flavonoid O-methyltransferase
Phe	Phenylalanine
PKC	Protein kinase C
PLA2	Phospholipase A2
PPi	Pyrophosphate
PRs	Pathogenesis-related proteins
ROS	Reactive oxygen species
SAH	Adenosyl-L-homocysteine
SAM	S-Adenosyl-L-methionine
SDR	Short-chain dehydrogenase reductase
SK	Shikimate kinase
*syn*-CPP	(*5S*,*9S*,*10R*)-Copalyl diphosphate
TDC	Tryptophan decarboxylase
TPA	12-O-Tetradecanoylphorbol 13-acetate
Try	Tryptophan
TYDC	Tyrosine decarboxylase
Tyr	Tyrosine
UBQ5	Ubiquinone 5
UV	Ultraviolet radiation
VEGF	Vascular endothelial growth factor
WBA	Whole blood aggregometer
WRC	World rice core collection
